# Integrated Renewable Production of Sorbitol and Xylitol from Switchgrass

**DOI:** 10.1021/acs.iecr.1c00397

**Published:** 2021-04-12

**Authors:** Guillermo Galán, Mariano Martín, Ignacio E. Grossmann

**Affiliations:** † Department of Chemical Engineering, University of Salamanca, Plz Caidos 1-5, 37008 Salamanca, Spain; ‡ Department of Chemical Engineering, Carnegie Mellon University, 5000 Forbes Avenue, Pittsburgh, Pennsylvania 15213, United States

## Abstract

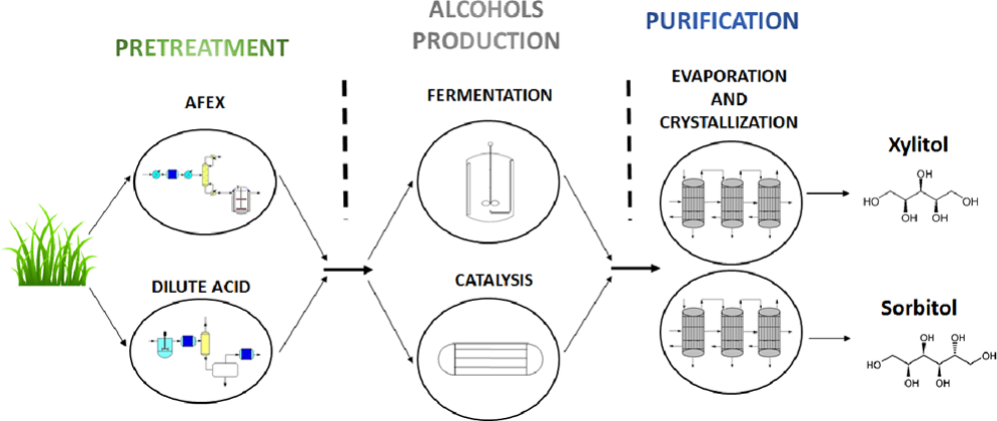

This work deals with the design of integrated facilities for the production of xylitol and sorbitol from lignocellulosic biomass. Xylitol can be obtained from xylose via fermentation or catalytic hydrogenation. Sorbitol is obtained from glucose, but preferably from fructose, and also via fermentation or catalytic hydrogenation. Fructose can be obtained from glucose via isomerization. Thus, a superstructure of alternatives is formulated to process switchgrass, corn stover, miscanthus, and other agricultural and forestry residues. Different pretreatments, such as dilute acid or ammonia fiber explosion (AFEX), for the fractionation of the biomass are evaluated. Next, after hydrolysis, the C5 and C6 sugars are processed separately for which a catalytic or a fermentation stage are considered. Glucose has to be isomerized before it can be processed. Finally, crystallization in a multistage evaporator system is used for purification. The optimization of the system is done by the use of dilute acid and the catalytic system. A system of 3 crystallizers is selected. For a facility that produces 145 kt/yr of xylitol and 157.6 kt/yr of sorbitol, the investment adds up to 120.74 M€ for a production cost of 0.28 €/kg products. The inverse engineering of biomass was also performed resulting in a composition of 15% water, 20% cellulose, 40% hemicellulose, 15% lignin, and 5% ash. The closest biomass corresponds to *Sargassum* (brown algae), which is capable of producing 230.5 kt/yr of xylitol and 116 kt/yr of sorbitol with investment and production costs of 120.5 M€ and 0.25 €/kg products, respectively.

## 1 Introduction

The chemical industry is undergoing a transformation toward a more sustainable future starting from the use of renewable instead of fossil resources, which constitutes the 7th principle of green chemistry.^1^ Biomass has emerged as a rich raw material in the production of energy and chemicals.^2^ While energy and fuels were the first focus of biorefineries design, such as first- and second-generation bioethanol,^3,4^ the valorization of biomass toward platform chemicals and added-value products is part of this new strategy. Lignocellulosic biomass is a promising feedstock as it consists of cellulose, hemicellulose, and lignin. Glucose is the building block of cellulose and can be used beyond the production of ethanol for the production of hydroxymethylfurfural,^5,6^*i*-butene,^7^ or sorbitol.^8^ The hemicellulose building block is xylan, the precursor of xylose that can be converted into furfural^6^ or xylitol among others. Apart from sweeteners, xylitol and sorbitol are considered in the production of dietetic foods to diabetic patients because of the non-insulin-dependent metabolic pathway. They can be used also in pharmaceutical applications (mainly as a carrier), the cosmetics industry (as an emulsion stabilizer), as a moisturizer, texturizer, and softener.^9^ The US Department of Energy^10,11^ lists xylitol and sorbitol as the top 12 high-value-added building block intermediate chemicals that can be produced from renewable biomass resources, while the EU has included them both as part of the map of potential value chains based on sugars^12^ due to the fact that there already exist commercial markets^13^ with the potential to replace petrochemicals^14^

The chemical synthesis of sorbitol has been evaluated from glucose via catalytic hydrogenation,^15,16^ or from the fermentation of fructose^17,18^ produced via glucose isomerization.^19,20^ In addition, technoeconomic studies have been performed to evaluate a biorefinery that uses lignocellulosic residues for the production of sorbitol, without considering the use of hemicellulose.^21^ Separately, the yield of xylitol synthesis has been evaluated via xylose fermentation^22^ as well as hydrogenation.^23^ The technoeconomic analysis comparing both synthetic paths has been presented by Mountraki et al.,^24^ while biorefineries based on sugarcane lignocellulosic materials toward the production of xylitol, citric acid, and glutamic acids have also been presented.^25^ However, lignocellulosic biomass contains the building blocks for the production of both products simultaneously, and so far no biorefinery has considered the production of both.

In this work, a mathematical optimization approach has been applied for the systematic comparison of synthesis routes for the simultaneous production of xylitol and sorbitol from biomass. The study allows optimizing the operating conditions of the different units by including surrogate models for all major transformations based on experimental data. The rest of the work is structured as follows. Section 2 presents the description of the superstructure of the alternatives. Section 3 describes the models developed for each one of the steps and technologies. Section 4 shows the solution procedure. In Section 5, the major results of the work are presented including the process design and the economic evaluation of all pretreatments and synthetic routes using switchgrass and biomass of agricultural and forestry origin. A cost comparison is also included using the biomass of optimal composition. Section 6 summarizes the conclusions of the work.

## 2 Overall Process Description

**Figure 1:**
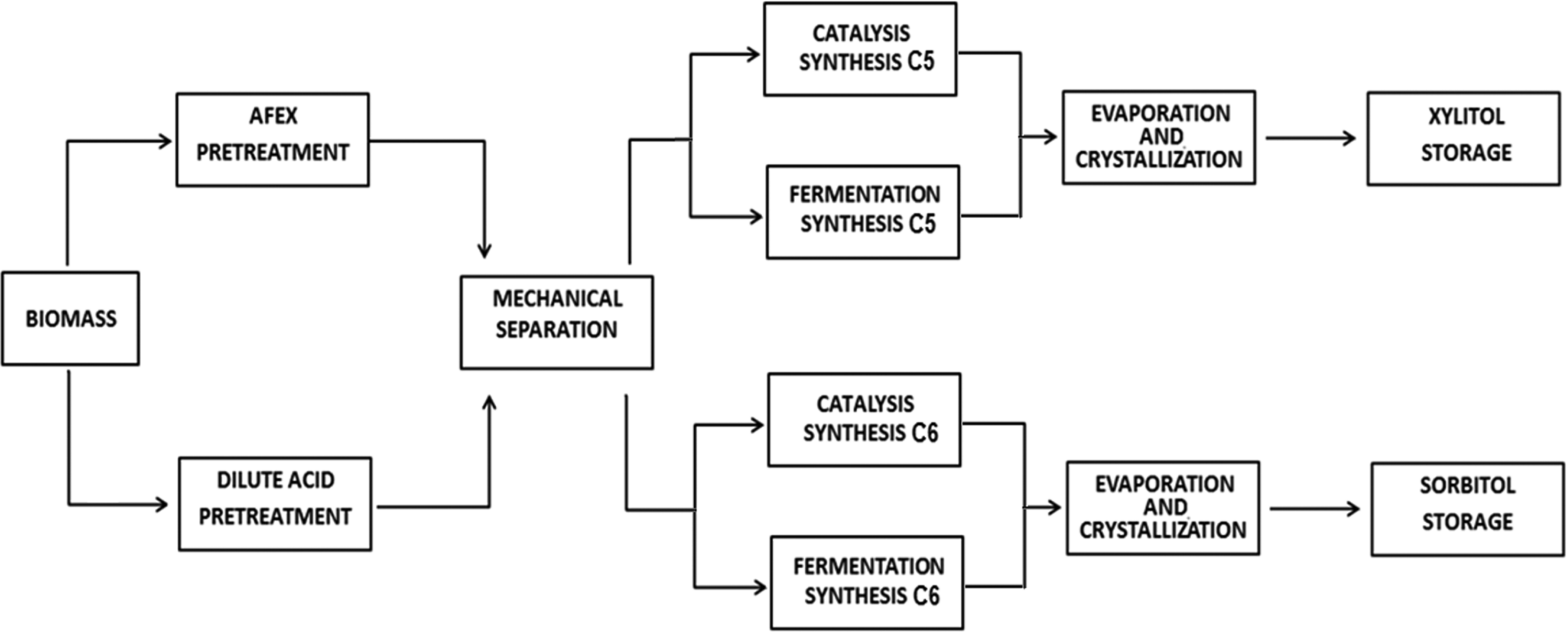
Superstructure for the renewable production of xylitol and sorbitol.

The superstructure used for process synthesis is shown in Figure 1. Biomass must follow a size reduction step before pretreatment, and there are a large number of alternative pretreatments.^2,26−28^ The ones more widely used are (1) steam explosion–dilute acid (H_2_SO_4_) pretreatment^1,29−31^ and (2) ammonia fiber explosion (AFEX).^27,32,33^ Sorbitol can be produced from glucose while hemicelluloses are used for the production of xylitol.^7^ Thus, once the lignocellulosic structure of the biomass is broken down, the cellulose and hemicelluloses are separated. Between both pretreatments, only dilute acid pretreatment allows releasing xylose from hemicellulose. The AFEX-pretreated biomass requires further hydrolysis at 50 °C. In this case, only xylan is hydrolyzed using an enzyme, xylanase, to promote the degradation. Similar considerations have been used in previous works, as shown by Aristizábal and Gomez.^34^ Cellulose has to be hydrolyzed at 45–50 °C for 3 days to obtain glucose.^30,35−37^

Glucose and xylose may follow two different pathways to produce sorbitol and xylitol: fermentation and catalytic synthesis. Xylitol can be produced via fermentation using the bacteria *Candida guilliermondii*,^38^ and adjusting the operating conditions at 30 °C and 1 bar of pressure with a residence time from 35 h to over 100 h.^24^ The fermentative production of sorbitol follows two steps: (1) an isomerization stage from glucose to fructose, which is carried out by *Streptomyces* sp. at 70 °C,^20^ and (2) fructose fermentation to sorbitol. The catalytic production of xylitol is performed in a three-phase stirred-tank reactor operating at 100–120 °C and 40–60 bar for 60–241 min.^39^ The reaction uses Ru as a catalyst supported generally over NiO, TiO_2_, activated carbon, or zeotype.^40−42^ Sorbitol production follows a similar route. The reaction is carried out also in a three-phase stirred reactor employing Ru-modified particles as a catalyst.^16,43,44^ The operating conditions are 100–140 °C and 40–60 bar^16^ for 60–240 min.

The purification process is performed using two parallel multieffect evaporator systems, one per product. For the final products to crystallize, the water is evaporated saturating the xylitol and sorbitol solutions. Commercial steam is used only in the first effect.

## 3 Mathematical Modeling

All of the operations in the production of renewable xylitol and sorbitol from switchgrass are modeled with mass and energy balances, experimental yields, thermodynamic and chemical equilibrium, and thumb rules.^45^ To model the pretreatments, surrogate models are developed using data from experiments or rigorous simulations of the units, ammonia recovery, and catalytic xylitol production.

The superstructure is mathematically formulated in terms of temperature, total and component mass flows, and component mass fractions. The components in the system are included in set *J* = {water, H_2_, H_2_SO_4_, CaO, ammonia, protein, cellulose, hemicellulose, glucose, xylose, lignin, ash, CO_2_, O_2_, cells, glycerol, succinic acid, acetic acid, lactic acid, gypsum, ethanol, xylitol, sorbitol}.

### 3.1 Pretreatment

The main objective of the pretreatment consists of breaking down the raw material. The challenge with lignocellulosic biomass is the complex plant structure. It consists of a matrix of lignin. Within this skeleton, there is a structure formed by cellulose and hemicellulose, polymers consisting mainly of glucose and xylose linked by *o*-glycosidic bonds. For the base case, switchgrass is considered as raw material, a native species in the eastern part of the United States. We can assume a typical composition to be as follows: 15–20% moisture, 25–40% cellulose, 20–30% hemicellulose, 15–25% lignin, and 5.95% ash. The feedstock is washed and its size is reduced by grinding.^2,46^ The washing and grinding stages are considered only in terms of energy consumption (162 MJ/t)^46^ and cost analysis since they do not change the composition of the feedstock. Next, the two alternative pretreatments, dilute acid pretreatment and AFEX, are analyzed comparing their yield toward structure degradation.^27,47−50^

#### 3.1.1 Ammonia Fiber Explosion (AFEX)

In this method, the lignocellulosic biomass is treated with ammonia solution at medium to high temperatures and high pressures to break the complex matrix of lignin. To avoid the possible environmental hazards and to reduce the costs of operation, it is necessary to recover ammonia. The slurry stream rich in water and polymers is sent to an enzymatic process to release the sugars.^27,32,33,51^ Garlock et al.^51^ evaluated the yield of this pretreatment for different species of switchgrass. The set of experiments developed studied the effect of the ammonia ratio (kg/kg of biomass), the water load, the operating temperature (°C), and the contact time (min) at 2.1 MPa on the yield of sugars. This operation is carried out in batch mode. To ensure continuous operation, additional reactors in parallel with storage tanks are required.^27,52^ The slurry containing ammonia is sent to a distillation column that operates typically at 3 bar and 140 °C to avoid ammonia decomposition.^29,53^ The pressure may be raised up to 5 bar as long as the amount of ammonia in the bottoms slurry is present in traces. In order to obtain the feed, reboiler, and condenser temperatures, as well as the purity and the recovery yield as a function of feed composition in ammonia and the operating pressure, a surrogate model developed in the previous work^54^ from a rigorous simulation of the column in ChemCAD is used. The recovered ammonia is absorbed into water, pressurized to a liquid, and recycled. This point is key toward the economic savings, avoiding the compression of ammonia gas. Only 0.5% of the total ammonia is lost in the slurry and can be used as a nutrient in the fermentation downstream.^52^ Ammonia recovered is thus fed to the system continuously.

**Figure 2:**
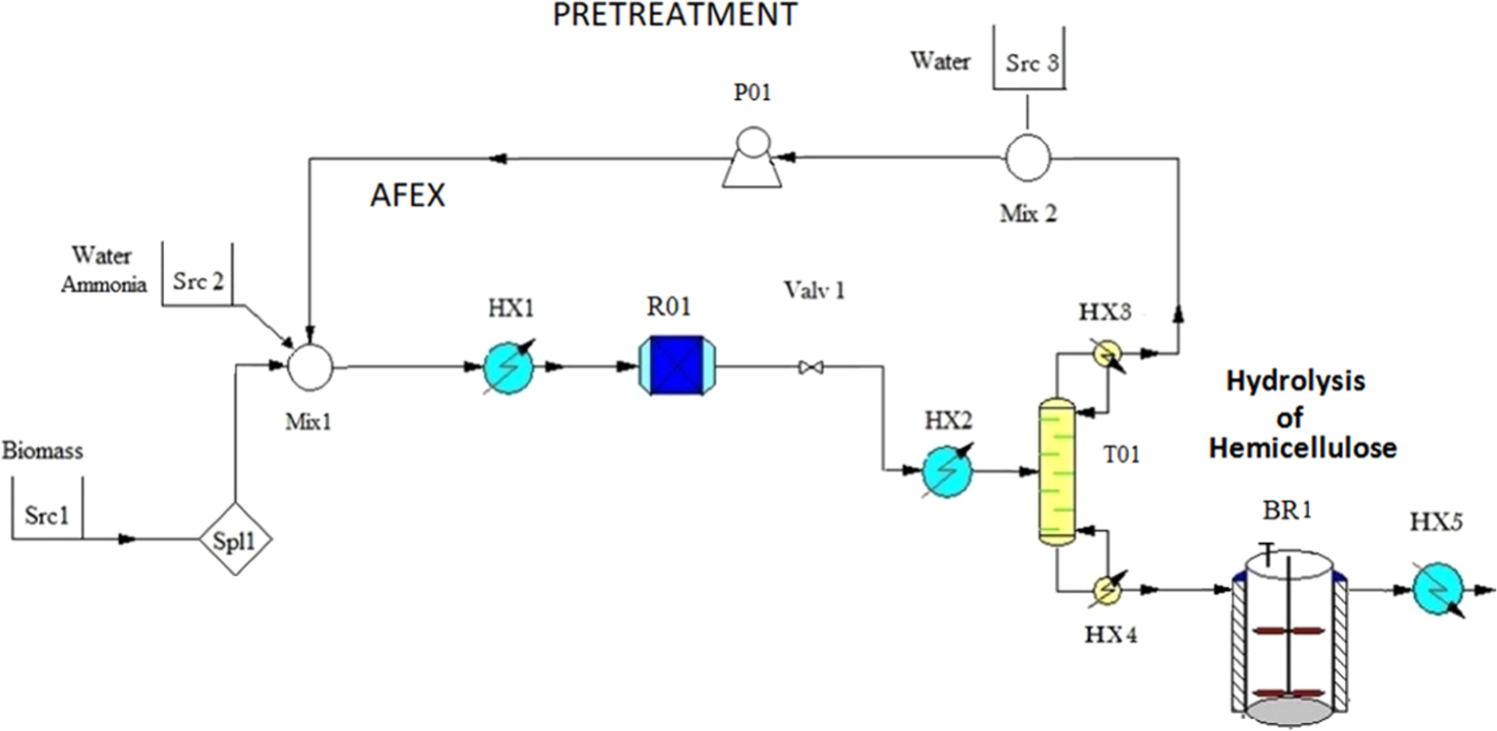
Schematic of AFEX pretreatment.

Next, based on experimental results, we consider that glucose monomer is available in the broth. The glucose monomer is generated at this stage but it will be hydrated in the hydrolysis stage to obtain glucose molecules; however, for the sake of reducing the number of components, dehydrated glucose is obtained and will be hydrated later on. Xylose is produced from the pretreated biomass via specific hydrolysis in BR1, as shown in Figure 2.^54^ The complete model and the operational conditions are summarized in the Supporting Information (AFEX Pretreatment).

#### 3.1.2 Dilute Acid

**Figure 3:**
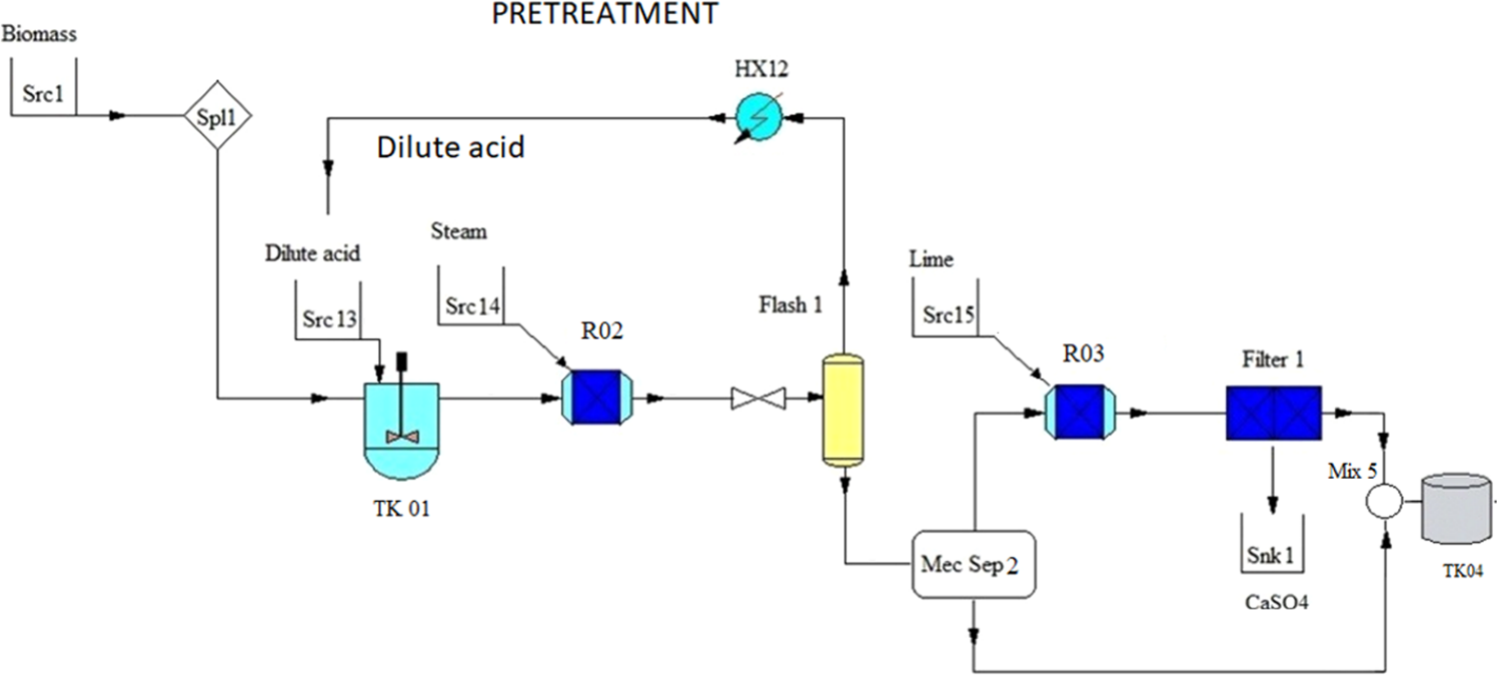
Schematic of dilute acid pretreatment.

This pretreatment uses sulfuric acid and steam explosion to degrade the lignocellulosic structure of the biomass; see Figure 3. Experimental data on the performance of this pretreatment are presented in the form of response surface models^55−57^ and mechanistic kinetics.^58^ The first approach is more convenient for process synthesis. The yield of sugars released from the biomass depends on the operating temperature, the concentration of the acid, the residence time, and the amount of enzyme used, per gram of glucan, in the hydrolysis stage.^57^ As in the previous case, the glucose monomer is generated at this stage but it will be hydrated in the hydrolysis stage to obtain the sugar molecules. Xylose obtained can be directly used in the catalytic or the fermentation process. Using the experimental data provided in Shi’s paper,^57^ the surface of response surrogates are developed to estimate the yield of the glucose and xylose released.^58^

Next, a flash (Flash 1) reduces the water content of the slurry, thereby reducing water consumption and providing energy to the process. The mechanical separation (Mec Sep 2) allows the separation of the slurry. The solid phase is bypassed and the liquid phase is neutralized in Reactor 3 (R03) using solid lime (CaO).^1,30,59,60^ Lime is a low-cost chemical, and the gypsum formed is easily separated from the liquid medium^61^ using a filtration stage (Filter 1). The optimal time for this reaction is in the range of 3–10 min. The neutralized liquid stream is mixed adiabatically in Tank 4 with the biomass, and the resulting slurry is sent for hydrolysis. The cellulose needs a further step before it can be broken down into glucose but the xylose is ready to be used.
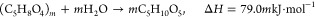
1The complete model and the operating conditions are summarized in the Supporting Information (Dilute Acid Pretreatment).

### 3.2 Xylitol and Sorbitol Production by the Fermentation Pathway

**Figure 4:**
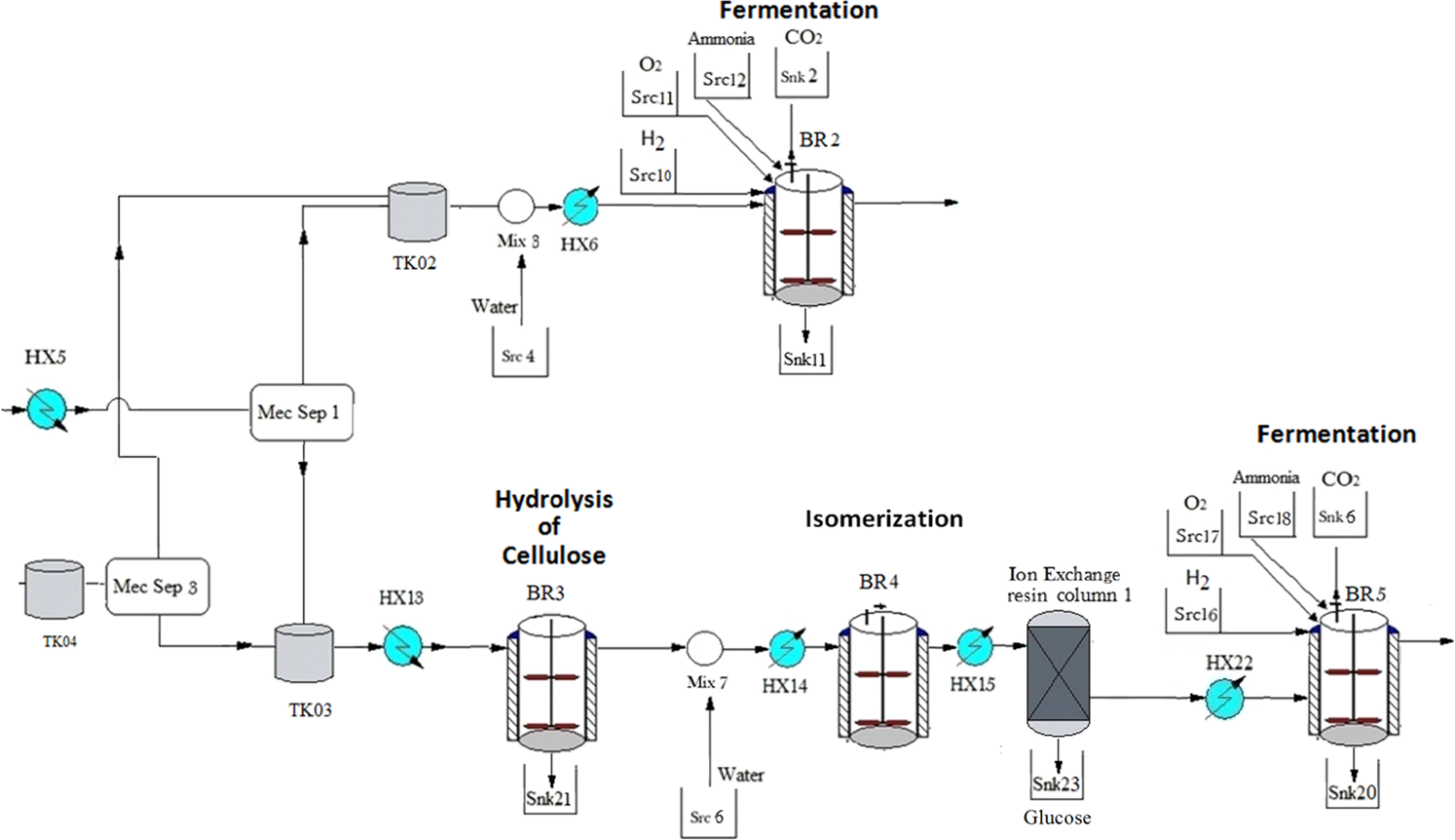
Details of the fermentation pathway.

The streams rich in xylose and glucose are mechanically separated using a centrifugation process, Mec Sep-1 and Mec Sep-3, depending on the pretreatment. After this process, two parallel streams are obtained, each of them with a different proportion of sugars; see Figure 4.

The production of xylitol from xylose fermentation is carried out in fermenter BR-2 by the bacteria, immobilized enzyme systems, or the fungus *C. guilliermondii* ^38^ adjusting the operating conditions to 30 °C and 1 bar pressure, with a residence time from 35 to 100 h.^24^ Heat exchanger (HX) HX-6 and pumps are used to control the temperature and pressure of this operation. The fermentation reaction is as follows
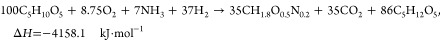
2During the process, other secondary reactions may also take place (as shown in eq 2) and are most important, which consume almost all of the xylose reaching a conversion of 92%.^24^ The unconverted xylose remains in the liquid phase. The hydrogen and oxygen needed are directly fed into the fermenter (Src-10 and Src-11, respectively). Ammonia as a nitrogen source will also be provided (from Src-4) in the form of an aqueous solution to avoid the temperature increase due to the large heat of mixing in BR-2 and to control the input xylose concentration to be in the range of 50–100 g/L.^24^ In this case, the optimal concentration is 100 g/L because it allows one to use the least amount of diluted water possible.

The production of sorbitol is carried out by fructose fermentation. The pretreatment releases glucose in the form of a dehydrated molecule. Then, glucose is formed by hydrolysis. The next operation consists of the isomerization to fructose^20^ by *Zymomonas mobilis* in its metabolic route to produce sorbitol.^17^ The optimization process of this step consists of optimizing the yield of glucose to fructose. The remaining glucose can be sold to obtain additional revenue but, generally, it is used as a nutrient for the microorganism, avoiding secondary reactions as well. Glucose isomerization is described by eqs 3–5.^20^
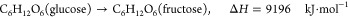
3
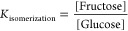
4
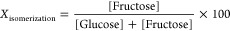
5The key factors in the isomerization are the temperature and the equilibrium isomerization constant, both related, and are shown in Table 1.^20^

**Table 1:** Range of Temperatures for Glucose Isomerization

*T* (°C)	*K*_isomerization_	Δ*H* (kJ/mol)
25	0.74	9196
40	0.92	9196
60	1.15	9196
70	1.3	9196

The increase in temperature has a direct effect on the increase in the equilibrium constant of isomerization. Therefore, higher operating temperatures result in higher glucose conversion, but Takasaki^20^ stipulates temperatures of 70 °C as an upper bound because it is the maximum temperature allowed for the bacteria *Streptomycessp*. Values beyond this limit give rise to secondary reactions that result in a decrease of the enzyme or bacterial activity.

The stream from the isomerization process is directed to an ion-exchange resin column (IER1), where selective separation of sugars takes place. A stream rich in fructose (>95%) is obtained.^22^ The glucose retained in the column resins can be recovered for reuse in the process or can be sold as high-purity glucose becoming a source of additional process revenue. The stream enriched in fructose is sent to a fermentation process (BR-5) where the sorbitol production reaction is carried out, as shown in eq 6.
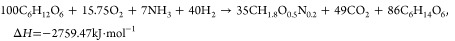
6As in xylose fermentation, it is also necessary to adjust the concentration of fructose in the fermentation medium to maximize its conversion. Chung et al.^17^ have studied the conversion of fructose to sorbitol by *Z. mobilis* as a function of fructose concentration. Using those data, a correlation between fructose conversion and its concentration has been developed (eq 7). The graphical representation can be seen in the Supporting Information (Xylitol and Sorbitol Production by Fermentation Pathway).
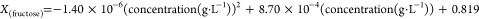
7The feasible concentration range of fructose is between 100 and 300 g/L, which is used to obtain a conversion above 90%. These conditions are necessary to determine the operating conditions in BR-5 since the fructose concentration determines the operation and size of the units downstream.

### 3.3 Xylitol and Sorbitol Production by the Catalytic Pathway

**Figure 5:**
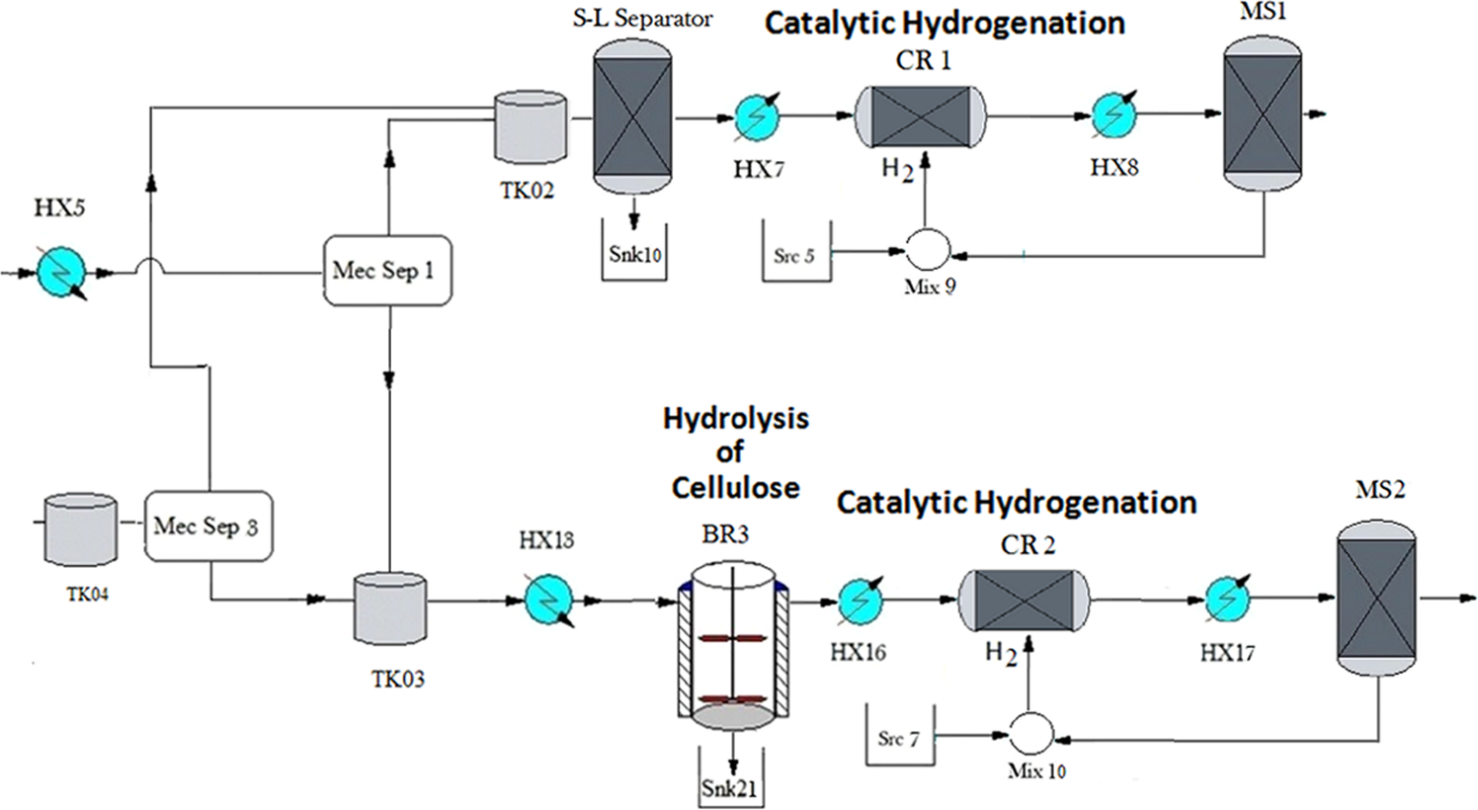
Catalytic pathway.

The streams rich in sugars can also follow a catalytic hydrogenation process in order to obtain xylitol and sorbitol; see Figure 5. On the one hand, the stream rich in xylose is directed to the solid–liquid separator (S–L separator). In this unit, lignin is removed, which is mainly used to obtain pellets that are used as a source of energy for the process; lignin is considered to be slightly wet after separation. We assume that the remaining hemicellulose is removed here along with lignin. The resulting stream is heated up to 100–120 °C in HX-07 and compressed up to 40–60 bar so that the catalytic hydrogenation reaction (eq 8) can be carried out in the reactor (CR-1) for 60–241 min.^39^
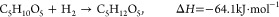
8The classical method to obtain xylitol from xylose is carried out in a three-phase stirred reactor employing Ni-Raney^62^ or supported Ru-modified particles as a catalyst.^39^ Ni-Raney is cheaper and has higher catalytic activity.^63,64^ But, the disadvantages of this type of catalyst are the leaching of nickel, fast deactivation, and nickel dissolution.^16,40,63^ Thus, catalysts based on supported Ru are selected because they have a slower deactivation rate and high selectivity. The supports are generally NiO, TiO_2_, activated carbon, or zeotype.^40−42^

The fresh hydrogen stream is fed to the reactor (CR-1) from the source (Src 5) at the same temperature and pressure as the xylose stream. An excess of hydrogen is fed to ensure complete conversion of xylose, and the membrane MS-1 is used. The excess of hydrogen is recovered and recirculated to the Mix-9 mixer.

To predict the yield of the reactor, experimental results from the literature^39^ have been used to develop a model for the conversion as a function of the reaction time. The shape of the profile is sigmoidal and the following equation (eq 9) is used.
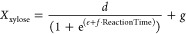
9The best fitting was developed using the Agustinson equation (eq 10).
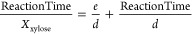
10This linearization model shows a good fit above 50% conversion. However, the operating conditions depend on the pressure and temperature. A two-stage fitting procedure is used to include the effect of pressure and temperature on the fitting parameters *d* and *e*, and they are summarized in Table 2.

**Table 2:** Fitting Parameters *d* and *e*

*P* (bar)	*T* (°C)	*d*	*e*
40	100	1.4154	87.2875
	110	1.0572	11.8766
	120	1.0045	2.5342
50	100	1.3168	66.3309
	110	1.0179	4.0116
	120	0.9916	0.0533
60	100	1.1258	26.1553
	110	1.0288	6.0803
	120	0.9869	0.8522

The objective of the previous fittings is to determine the effect of the operating conditions on parameters *d* and *e* of the linearization model and to create a model that allows predicting the optimal operating conditions for the catalytic hydrogenation of xylose. The fitting of these parameters is obtained based on parabolas, shown in eqs 11 and 12.
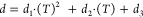
11
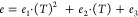
12For each pressure, the adjustment coefficients, *d*_1_ and *e*_1_, *d*_2_ and *e*_2_, and *d*_3_ and *e*_3_, corresponding to the quadratic, linear, and independent terms, respectively, are obtained (summarized in Table 3). The fitting coefficients are shown in the Supporting Information (Parameters Fitting, Figures S7–S12). The adjustment coefficients *d*_1_, *d*_2_, and *d*_3_ are summarized in Table 3.
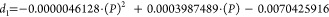
13
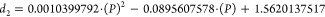
14
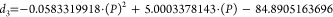
15The coefficient values *e*_1_, *e*_2_, and *e*_3_ are summarized in Table 4.
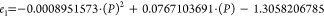
16
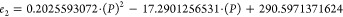
17
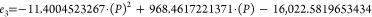
18On the other hand, the stream rich in glucose is directed toward heat-exchanger HX-16, adjusting the temperature between 100–140 °C and the pressure to 40–60 bar^16^ before directing it to the catalytic reactor CR-2. The reaction is run for 60–240 min. The synthesis of sorbitol from glucose is carried out in a three-phase stirred reactor employing Ru-modified particles as a catalyst.^16,43,44^ The catalysts based on Ni-Raney allow achieving a high conversion of glucose but have the same disadvantages as the ones for the production of xylitol.
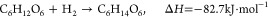
19A fresh stream of H_2_ at the reaction temperature and pressure is fed from Src-7 to CR-2. It is fed in stoichiometric proportions according to eq 19, but an excess of H_2_ atmosphere is maintained, which is constantly recovered through the membrane MS-2. As in the case of the production of xylitol production, the excess hydrogen is recovered and recycled to Mix-10. Due to the lack of a profile of the evolution of glucose conversion with respect to temperature and pressure, the modeling of the catalytic reactor and the optimal operating conditions were based on the data reported on the conversion of glucose (above 99.9%),^44,60,65^ and maximizing the amount of sorbitol produced simultaneously minimizing the costs associated with energy (see Section 3.4).

**Table 3:** Fitting Coefficients *d*_1_, *d*_2_, and *d*_3_

*P* (bar)	*d*_1_ (bar^–2^)	*d*_2_ (bar^–1^)	*d*_3_
40	0.0015268518	–0.3564497953	21.791809307
50	0.0013628024	–0.3160760678	19.2963948214
60	0.0002761889	–0.0677064946	5.1345819739

**Table 4:** Fitting Coefficients *e*_1_, *e*_2_, and *e*_3_

*P* (bar)	*e*_1_ (min/bar^2^)	*e*_2_ (min/bar)	*e*_3_
40	0.3303424081	–76.912997503	4475.1631974317
50	0.291804531	–67.5108775881	3899.3733247795
60	0.0742351944	–17.5968962407	1043.4929867889

Two heat exchangers, HX-8- and HX-17, with auxiliary utilities, control the temperature of the streams that are directed to the membrane modules in cases they have a temperature above the allowed one.

### 3.4 Xylitol and Sorbitol Purification

**Figure 6:**
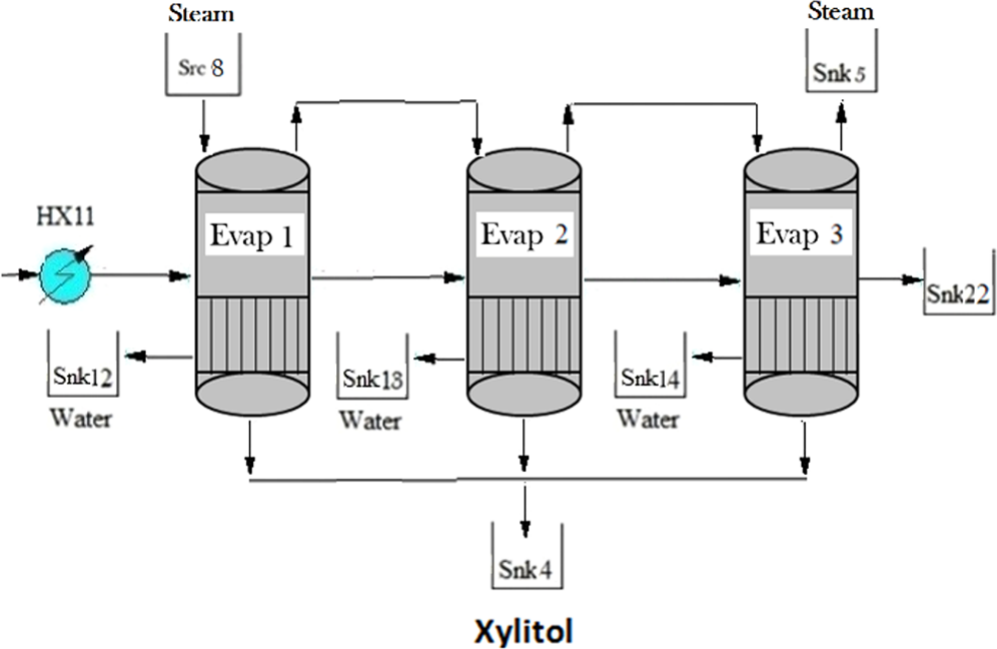
Xylitol purification.

**Figure 7:**
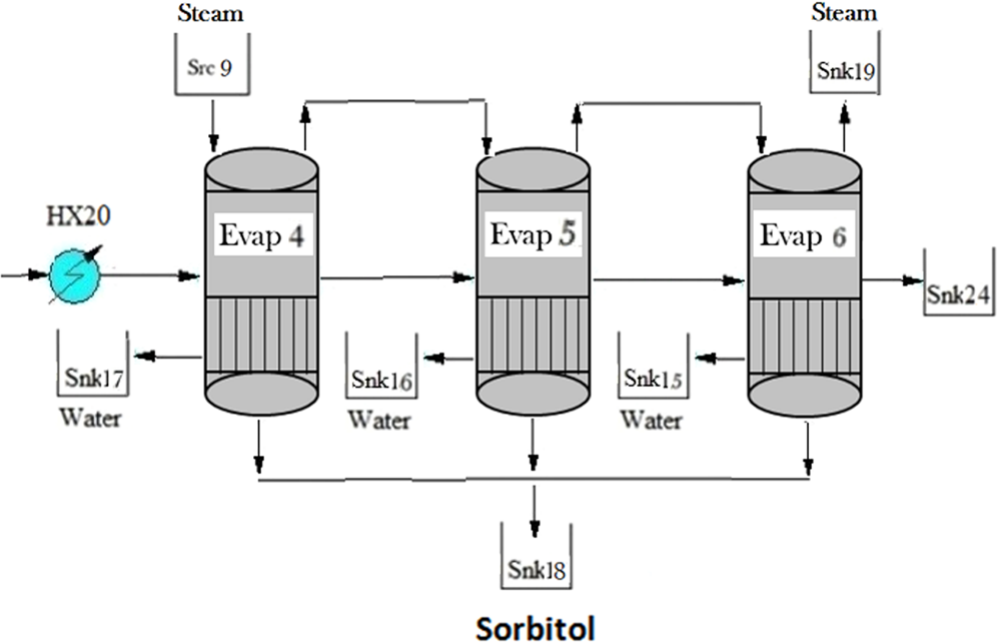
Sorbitol purification.

Purification of xylitol and sorbitol is carried out using two sets of multieffect evaporators, Evap1-Evap2-Evap3 and Evap4-Evap5-Evap6, for xylitol and sorbitol, respectively; see Figures 6 and 7. The streams coming from the MS-1 and MS-2 membranes, rich in xylitol and sorbitol, are directed toward HX-11 and HX-20 where their temperatures are set depending on the solubility of different sugars (eqs 22–25) to improve the purification process.

The operation of multieffect evaporators is based on the use of commercial steam in the first one with the aim of evaporating water from the solution, and producing steam that is used as a heating agent in the next effect. The mass balance of the solute is as follows^66^
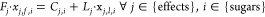
20
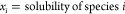
21where *x* is given by eqs 20–25 for each of the sugar and sugar alcohols.
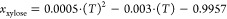
22
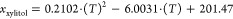
23
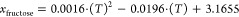
24
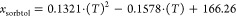
25The mass balance of water in the evaporation chamber of each effect is given by eq 26.
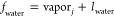
26This condition is common for all sets of evaporators because the steam used in each effect is not in direct contact with the sugar solutions. The energy balance for the first effect is given by eq 27
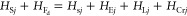
27while for the other effects, the balance is as in eq 28.
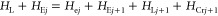
28The enthalpies of the streams are calculated by the components considering enthalpies of formation, crystallization, and solution of the solids. For the rest of the liquid streams, they can be calculated using eqs 29 and 30.
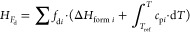
29where
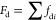
30

*H*_E_ is the enthalpy of the superheated steam since it is generated in a solution where sugar concentration increases.
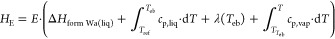
31For the rest of the vapor streams, it can also be calculated using eq 31. *H*_cr_ is the enthalpy of the crystals and can be calculated by eq 32
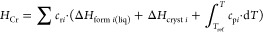
32The model is formulated so that the solutions must go from one effect to the next saturated in the sugar to be recovered. For this, the constraint given by eq 33 is used.
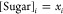
33Equation 33 introduces a relevant term in the calculation since the operating conditions change depending on the solubility of the sugar. The model shows different solutions depending on the chemical route. The catalytic synthesis process presents almost 100% conversion of xylitol and sorbitol. In this case, xylitol and sorbitol are free from impurities, allowing easier separations. However, fermentation results in incomplete conversion of sugars where some amounts of xylose and fructose are swept downstream. Sorbitol is obtained from fructose, but each one has a different solubility, which allows recovering them separately without the risk of having impurities. In the case of xylitol, it should be noted that as its solubility is higher than the solubility of xylose, the presence of a certain amount of xylose in solution results in either lower recovery of xylitol or a decrease in its purity.

The temperature of the evaporating chamber is calculated using the ebullioscopy increment produced by the presence of the sugars in the solution
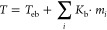
34
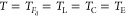
35where *K*_b_ is the boiling constant of water (equal to 0.512 kg/(mol K)) and *m*_*i*_ is the molality of each sugar molecule. The pressure of each chamber is given as a function of *T*_eb_ using the Antoine′s equation (eq 36)
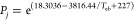
36The additional process constraints are given by eqs 37 and 38.
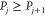
37
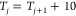
38

## 4 Solution Procedure

For simplicity, due to the presence of only one binary variable, the one related to the selection of the pretreatment, two nonlinear optimization models (NLPs) are solved involving 2700 equations and 3800 variables each. The major decision variables are the operating conditions at the pretreatment reactors, feed ratios and operating temperatures, the operating conditions during the synthesis, the split fraction, and operating pressures and temperatures at the evaporator sets. The last one depends on the yield of each pretreatment and the biomass composition. The model is solved using a multistart optimization approach in GAMS^(R)^ with CONOPT 3.0 as the preferred solver. The objective function is the maximization of a simplified profit including xylitol and sorbitol production, and the thermal energy and hydrogen consumed due to the fact that it is the largest variable cost (eq 39)
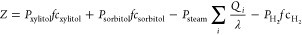
39Next, a heat exchanger network is developed^67^ and the economic evaluation is performed to compute production and investment costs.^68^ The production cost involves annualized equipment, chemicals (enzymes, sulfuric acid, CaO, ammonia, and the profit from gypsum), labor, utilities, and raw materials. The costs for utilities are updated from the literature: steam, 19 $/t; cooling water, 0.057 $/t; electricity, 1.7 × 10^–8^ $/J;^69^ and the base price for biomass: 100 €/t. The estimation of investment is performed with the factorial method.^68^ First, the equipment cost is estimated with the mass and energy balances obtained from optimization. The cost for the equipment such as heat exchangers, fermenters, tanks, distillation column, mechanical separation, filters, and molecular sieves is updated from the values calculated using the correlations developed by the authors; see Supporting Information of Martín and Grossmann^3^ and Almena and Martín.^70^ Next, the equipment cost is calculated as a function of the equipment cost, using factors of 3.15 and 1.4. These factors correspond to the facility processes, fluids and solids for the physical, and total fixed costs.^68^

The economic study is followed by the analysis of the operation of different biomass types toward the production of xylitol and sorbitol.

## 5 Results

The facility is based on a feed of 18 kg/s of biomass, typically used in bioethanol production facilities and biomass processing^71^ using switchgrass as a base case.

### 5.1 Facility Operation

Tables 5–7 summarize the operating conditions of the pretreatments and synthesis paths, and the purification of xylitol and sorbitol carried out in the evaporators.

**Table 5:** Pressure and Temperature for the Pretreatments

	dilute acid-catalysis hydrogenation		AFEX-catalysis hydrogenation		dilute acid-fermentation		AFEX-fermentation	
	*P* (bar)	*T* (°C)	*P* (bar)	*T* (°C)	*P* (bar)	*T* (°C)	*P* (bar)	*T* (°C)
reactor 1			21	109.8			21	109.3
reactor 2	1	180			1	180		
reactor 3	1	107.4			1	105.4		
hydrolysis of hemicellulose (BR1)					1	50	1	50
hydrolysis of cellulose (BR3)	1	50	1	50	1	50	1	50

**Table 6:** Pressure and Temperature for the Synthesis

	dilute acid-catalysis hydrogenation		AFEX-catalysis hydrogenation		dilute acid-fermentation		AFEX-fermentation	
	*P* (bar)	*T* (°C)	*P* (bar)	*T* (°C)	*P* (bar)	*T* (°C)	*P* (bar)	*T* (°C)
catalytic reactor CR1	46.86	104.43	47.92	100				
catalytic reactor CR2	46.87	100	47.9	100				
fermenter BR2					1	30	1	30
fermenter BR4					1	70	1	70
fermenter BR5					1	30	1	30

**Table 7:** Pressure and Temperature for the Evaporators

		dilute acid-catalysis hydrogenation		AFEX-catalysis hydrogenation		dilute acid-fermentation		AFEX-fermentation	
		*P* (mmHg)	*T* (°C)	*P* (mmHg)	*T* (°C)	*P* (mmHg)	*T* (°C)	*P* (mmHg)	*T* (°C)
xylitol	evap1	119.31	55.24	112.92	54.10	152.99	60.53	161.04	60.53
	evap2	108.88	53.35	107.27	53.04	145.34	59.42	145.34	59.42
	evap3	52.67	39.15	48.92	37.78				
	kg_steam_/kg_xylitol_	0.23		0.20		7.17		7.15	
sorbitol	evap4	148.7	59.91	145.11	59.39	152.79	60.50	151.81	60.36
	evap5	141.26	58.81	137.86	58.29	145.15	59.39	144.22	59.26
	evap6	133.06	57.54	126.07	56.40	123.26	55.92	121.56	55.63
	kg_steam_/kg_sorbitol_	1.09		0.76		2.76		2.26	

Regarding the pretreatments conditions, the dilute acid process requires a higher temperature than AFEX, 180 vs 110 °C, which together with the presence of the acid allows the degrading of a higher amount of hemicellulose to xylose. However, note that the acid can dehydrate sugars into furfural and furans, which are inhibitors for fermentation; we assume that it is not the case here.^54^ AFEX pretreatment requires higher pressure to break down the lignocellulosic structure of the biomass, and an additional stage for the hydrolysis of the hemicellulose that is carried out in a fermenter at 50 °C and 1 bar.

Within the synthesis routes, the catalytic process makes use of high pressures, close to 48 bar, and moderate temperatures, 100 °C or higher. The target of minizmizing energy consumption and increasing xylose and glucose conversion allow operations at or near the lower limit of the operating conditions.^16,39^ The fermentation process operates at mild pressure and temperature conditions, typically at 1 bar in all cases, and 30 °C for the xylose and fructose fermentations and 70 °C for the isomerization of glucose to fructose. Fermentation results in obtaining lower sugar conversion rates due to the metabolism of *C. guilliermondii* ^38^ and *Z. mobilis,*(17) larger volumes of feed, and an increase in the processing time.

The optimal operating conditions for each of the four alternative processes show the use of vacuum pressures to reduce the amount of commercial steam. Since all the processes work under approximately the same conditions of pressure and temperature, a comparison among the alternatives is performed based on the following ratios: kg_steam_/kg_xylitol_ and kg_steam_/kg_sorbitol_ (Table 7). The catalytic synthesis processes use a significantly smaller amount of steam in the evaporators due to higher concentrations of xylitol and sorbitol. These processes show higher conversions of glucose and xylose. In addition, the fermentation synthesis processes require large volumes of cell cultures, which results in lower conversions of sugars and larger amounts of commercial steam. The difference in the operating conditions is also due to the pretreatment yield. The dilute acid pretreatment has higher production rates of cellulose and hemicellulose than the AFEX pretreatment. As a result, the yields of xylitol and sorbitol are higher, which increases the product concentration in the streams. Thus, the boiling point of the mixture increases, requiring the use of more steam in the evaporators since the operating pressure should not be further reduced. In addition, the fermentation synthesis processes require an appropriate concentration of xylose.^38^ To achieve this, it is necessary to dilute the stream with water, which is later removed in the evaporators.

Table 8 shows the main operating ratios used to compare the alternative production paths. It can be observed that the yields of xylitol and sorbitol are lower in the fermentation paths due to the lower conversion achieved by the bacterial cultures. The processes with lowest operating to obtain xylitol and sorbitol are designed using the catalytic path. Within the catalytic process, the dilute acid pretreatment allows obtaining a higher concentration of sugars than the AFEX pretreatment, resulting in higher ratios of xylitol and sorbitol per kilogram of biomass and requiring less steam in their purification.

**Table 8:** Major Yields of the Alternative Production Paths^a^

	dilute acid-catalysis hydrogenation	AFEX-catalysis hydrogenation	dilute acid-fermentation	AFEX-fermentation
X_xylose_	1	1	0.92*	0.92*
X_glucose_ or X_fructose_	>0.999 (G)*	>0.999 (G)*	92.9 (F)	0.941 (F)
kg_H_2__/kg_biomass_	0.007	0.005	0.001	0.001
kg_H_2_SO_4__/kg_biomass_	0.024		0.017	
kg_NH_3__/kg_biomass_		1.052		1.052
kg_xylitol_/kg_biomass_	0.259	0.187	0.166	0.120
kg_sorbitol_/kg_biomass_	0.282	0.231	0.142	0.102
kg_steam_/kg_biomass_	0.505	0.587	2.013	1.374
*E* (kg_CO_2__/kg_biomass_)	0.145	0.136	0.296	0.206

a Abbreviations: F, from fructose; G, from glucose; *indicates from the literature.

The steam consumption in the hydrogenation-based processes is one-third to one-fourth of that consumed in the fermentation processes. However, hydrogen consumption is 5–6 times higher. An environmental index to account for both contributions simultaneously based on the equivalent CO_2_ emissions has been computed as follows: eq 40, where EF is the individual environmental factor related to each of the contributions, which is 10.5 kg CO_2_/kg H_2_^72^ and 0.142 kg CO_2_/kg steam,^73^ respectively. Note that the feedstock is biomass, a carbon-neutral source, and the index is built for comparison purposes among the different alternatives; the results are shown in Table 8. The larger energy consumption in the fermentation-based processes results in higher emissions for both pretreatments. In addition, between dilute acid and AFEX, the lower hydrogen consumption shows a lower impact due to the lower production of xylitol and sorbitol. Note that the emissions related to hydrogen correspond to the ones when it is not produced using renewable resources.
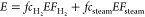
40

### 5.2 Economic Evaluation

**Figure 8:**
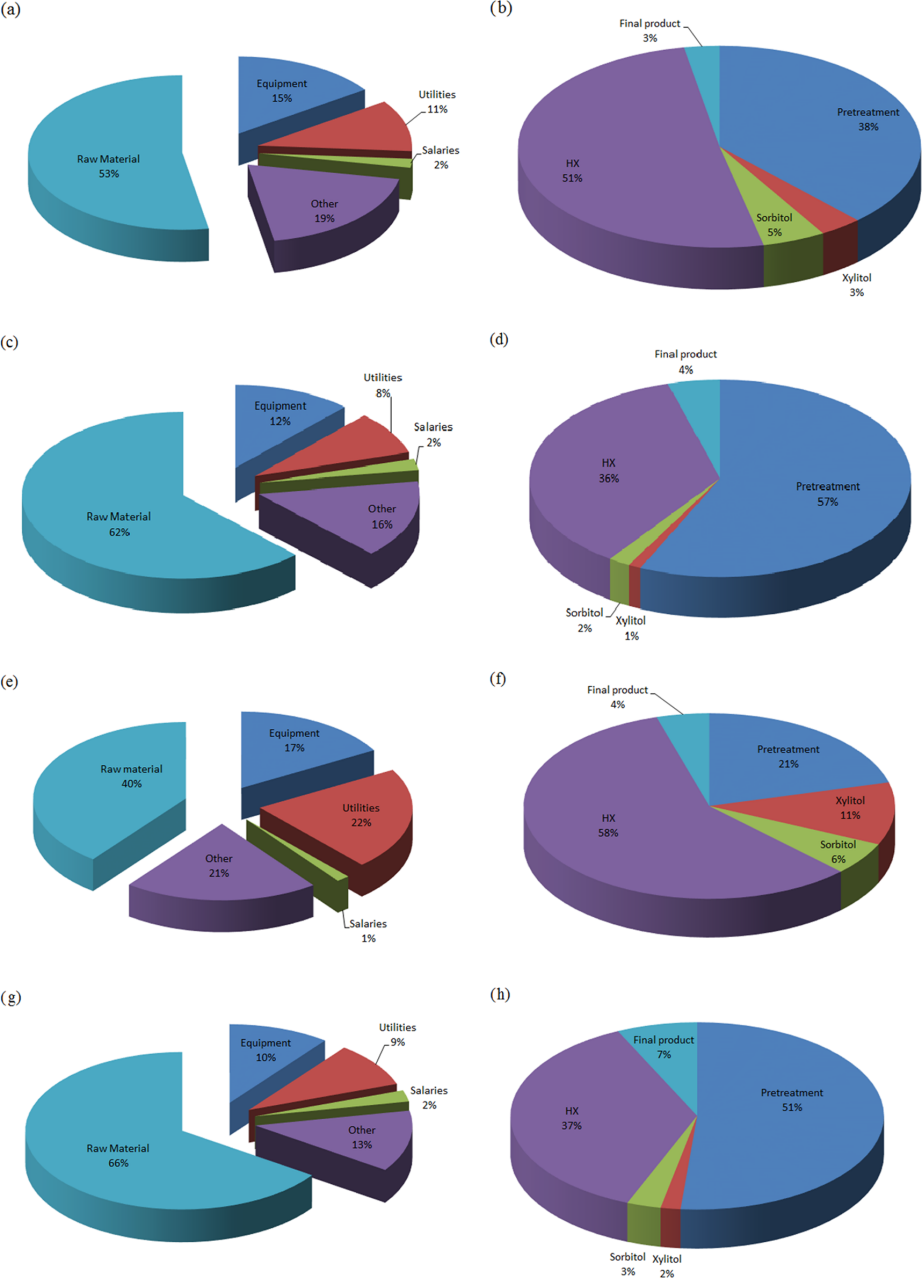
Detailed productions costs and investment costs for AFEX-fermentation (a and b), AFEX-catalytic hydrogenation (c and d), dilute acid-fermentation (e and f), and dilute acid-catalytic hydrogenation (g and h).

The economic evaluation is performed for both pretreatments and both synthesis processes, obtaining four alternative production paths, computing the production and investment costs. The detailed investment and production costs for the alternative production paths are shown in Figure 8. Table 9 summarizes the total costs, which increase when the fermentation paths are selected. In general, the fermentation process in continuous operation requires several fermenters operating in parallel. This, together with the need for larger volumes of flows to be treated and larger needs for steam used in the evaporators to concentrate the streams (Tables 7 and 8), results in larger costs. Regarding the pretreatments, AFEX involves higher costs than the dilute acid pretreatment in the case of catalysis hydrogenation as a distillation column is required to recover the ammonia used and an additional stage of hemicellulose hydrolysis, which explains the difference of 57 and 51% in the pretreatment costs between AFEX and dilute acid catalytic hydrogenation (Figure 8d,h). The fermentation paths feature the opposite behavior. This can be explained as follows: the increase in the amount of sugar results in the use of more water to adjust the xylose concentration in the fermenter, requiring more fermenters and a higher amount of commercial steam. This can be seen comparing the investment costs of heat exchangers (HX), which reach 46 and 58% for AFEX and dilute acid pretreatments, respectively (Figure 8b,f). This can also be observed in the percentage of utilities of production costs, reaching 18 and 22% of the total, respectively (Figure 8a,c). From Figure 8b,d,f,h, it can be concluded that dilute acid pretreatment is cheaper than AFEX pretreatment.

**Table 9:** Investment and Production Costs of the Alternative Production Paths

	dilute acid-catalysis hydrogenation	AFEX-catalysis hydrogenation	dilute acid- fermentation	AFEX- fermentation
Investment (M€)	120.8	146.4	323.7	273.5
Production cost (M€/yr)	18.7	22.9	45.8	37.8

Based on the major yields (Table 8) and lower investment and production costs (Table 9), the lowest cost process to obtain xylitol and sorbitol from switchgrass is the one that uses dilute acid as pretreatment and catalytic hydrogenation as a synthetic path.

### 5.3 Biomass Design and Evaluation

#### 5.3.1 Evaluation of Different Raw Materials

Once the dilute acid and catalytic synthesis are selected as the best pretreatment and synthetic paths, the analysis developed for switchgrass is also used to evaluate other typical biomasses such as corn stover, sugar bagasse, wheat straw and forest residues like a birch, pine and spruce, hybrid poplar. The results are summarized in Table 10 with the composition of each biomass. The composition of water has a direct effect on the steam ratio used; the higher the amount of water, the higher the amount of steam required to adjust the xylitol and sorbitol concentration. Furthermore, the increase in the fraction of water implies an increase in the amount of sulfuric acid used in the pretreatment. In the same way, the proportions of xylitol and sorbitol increase with a larger composition of hemicellulose and cellulose in the biomass, demanding a larger consumption of hydrogen. The environmental index for all of these biomasses remains in the range of 0.130–0.150 kg CO_2_/kg_biomass_ because of the use of the same production process. The fermentation paths used increase those values to above 0.20 kg CO_2_/kg_biomass_. In general, the amount of lignin plays a fundamental role in the costs since it is the biomass that cannot be transformed into products. In addition, an increase in the lignin content implies a higher energy consumption in the pretreatment and an increase in production and investment cost, in spite of the possible production of energy out of it. This energy is estimated considering a boiler efficiency of 75 and 26%,100 kJ per kilogram of lignin as an average value of heat of combustion. To be on the safe side, the credit out of this energy has not been included in the economic analysis. Based on these criteria and analyzing the data from Table 10, the biomass that offers the best results is corn stover, which is widely available in large parts of the world.

**Table 10:** Major Yields, Investment, and Production Costs for Different Biomasses^a^

	composition (%)														
biomass	W	C	HC	L	A	production cost (M€/yr)	investment cost (M€)	product cost (€/kg)	kg_xylitol_/kg_biomass_	kg_sorbitol_/kg_biomass_	kg_H_2_SO_4__/kg_biomass_	kg_H_2__/kg_biomass_	kg_steam_/kg_biomass_	energy from lignin (kW)	*E* (kg_CO_2__/kg_biomass_)
switchgrass	18.62	31.98	25.15	18.40	5.85	18.7	120.8	0.283	0.259	0.282	0.024	0.007	0.505	67.495	0.145
corn stover	16.95	41.05	31.39	6.34	4.27	16.3	111.7	0.220	0.323	0.361	0.023	0.009	0.386	25.663	0.146
birch (forest residue)	3.80	43.90	28.90	20.20	3.20	19.2	124.8	0.225	0.297	0.386	0.021	0.009	0.350	74.228	0.140
pine (forest residue)	5.00	40.70	26.90	27.00	4.00	20.4	129.6	0.244	0.323	0.358	0.021	0.008	0.353	97.973	0.134
spruce (forest residue)	2.00	42.00	27.30	27.40	1.30	20.5	130.1	0.238	0.281	0.370	0.023	0.008	0.347	99.421	0.135
hybrid poplar	6.91	50.80	26.20	15.50	5.90	18.4	121.5	0.213	0.270	0.447	0.022	0.009	0.357	57.374	0.144
sugar bagasse	7.00	41.00	30.10	21.20	7.00	19.4	125.5	0.230	0.310	0.361	0.022	0.008	0.361	77.889	0.140
wheat straw	8.43	40.26	30.56	16.52	4.23	18.5	121.5	0.228	0.315	0.354	0.022	0.008	0.364	61.446	0.140

a Abbreviations: W, water; C, cellulose; HC, hemicellulose; L, lignin; A, ash.

#### 5.3.2 Biomass Design

**Figure 9:**
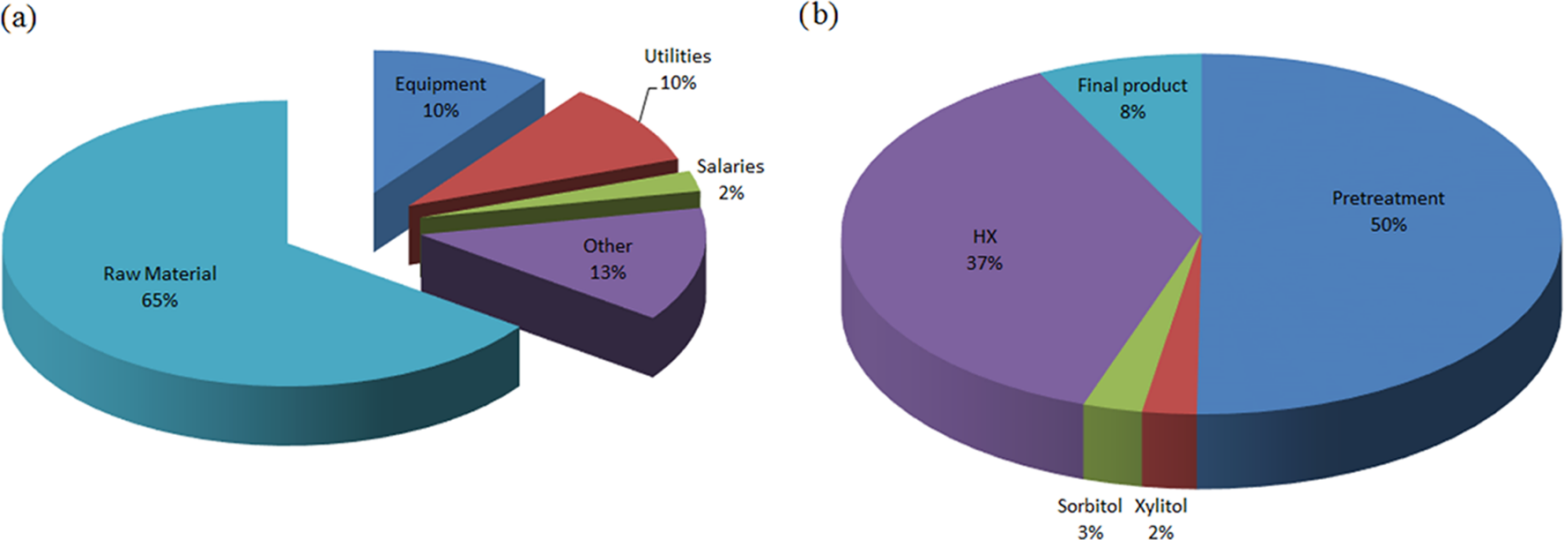
Dilute acid-catalytic hydrogenation free composition: productions costs (a) and investment costs (b).

As a complementary objective of this work, instead of using a fixed biomass composition, belonging to a lignocellulosic feedstock such as switchgrass, the optimal flowsheet is used to determine the best composition within the typical ranges of hemicelluloses, cellulose, and lignin for simultaneous production. The resulting composition is compared with a database to determine the most suitable biomass. In this way, the resulting biomass composition corresponds to 15% water, 20% cellulose, 40% hemicellulose, 15% lignin, 5% ash and 5% others. One that fits the best is sargassum algae (*sargassaceae*) with a composition of 20.48% cellulose and 43.19% hemicellulose. The production and investment costs for this biomass are 18.4 M€/yr (0.25 €/kg) and 120.5 M€, respectively. The breakdown in the costs is shown in Figure 9. Table 11 reports the major values for switchgrass and sargassum algae with slightly better values for the latter.

**Table 11:** Comparison of Investment and Production Costs between Switchgrass and Sargassum Algae

	dilute acid-catalysis hydrogenation (switchgrass)	dilute acid-catalysis hydrogenation (sargassum algae)
Investment (M€)	120.8	120.5
Production cost (M€/yr)	18.7	18.4
xylitol (kg/kg_biomass_)	0.26	0.41
sorbitol (kg/kg_biomass_)	0.28	0.21

To determine if this biomass is more promising than switchgrass, we compare the production and investment costs (Table 11). It can be seen that both biomasses yield similar values. The larger difference between them is the ratio of xylitol and sorbitol produced per kg of biomass. The amount of xylitol and sorbitol produced are 0.26 and 0.28 kg per kg of biomass for switchgrass, while in the case of the sargassum algae, values of 0.41 and 0.21 are obtained, respectively. The difference in the market prices of xylitol and sorbitol (3900 and 650 $/ton)^74^ suggests choosing a larger quantity produced of the product with a higher selling price. However, the environmental impact assessment plays an important role because it allows determining the process with the lowest CO_2_ emissions, and therefore, the one that uses the least amount of hydrogen and steam. Thus, the key parameters are the larger amount of xylitol produced per kg of biomass and the environmental index. The differences also explain the choice of sargassum algae as the best biomass, and not the corn stover (Table 12).

**Table 12:** Comparison of Xylitol and Sorbitol Production among Switchgrass, Sargassum Algae, and Corn Stover

	dilute acid-catalysis hydrogenation (switchgrass)	dilute acid-catalysis hydrogenation (sargassum algae)	dilute acid-catalysis hydrogenation (corn stover)
xylitol production (kt/yr)	145	230	181
sorbitol production (kt/yr)	157.6	116	202
xylitol (kg/kg_biomass_)	0.26	0.41	0.32
sorbitol (kg/kg_biomass_)	0.28	0.21	0.36
*E* (kg_CO_2__/kg_biomass_)	0.145	0.141	0.146

## 6 Conclusions

Xylitol and sorbitol production from the lignocellulosic biomass has been evaluated within the integrated biorefinery concept. Four different chemical paths are considered for the production of xylose and glucose and the final products. The biorefinery is modeled using first principles and surrogate models for each of the operations. The selected option is dilute acid as the pretreatment and catalytic hydrogenation as the synthetic path. Assuming that no inhibitors are produced for a facility that produces 145 kt/yr of xylitol and 157.6 kt/yr of sorbitol, the investment adds up to 120.8 M€ for a production cost of 0.28 €/kg. Integrated facilities operate at their optimum for specific biomass compositions. This framework also allows evaluating the best use of each biomass depending on its composition, as long as the models for the pretreatments are valid. Within the biomasses considered, corn stover is chosen as the best option, resulting in a production capacity of 181 kt/yr of xylitol and 202 kt/yr of sorbitol, while the investment adds up to 112 M€ for a production cost of 0.22 €/kg.

As a complementary study, the design of the optimal biomass was performed. Among all of the components, hemicellulose and cellulose are selected because they are the sources of sugars. Thus, the optimal biomass is the one that provides the closest composition with the existing biomass and provides a lower environmental index. For this case, the optimal composition of those components obtained was 20% cellulose and 40% hemicellulose, finding the closest composition of 20.48% cellulose and 43.19% hemicellulose in the sargassum algae (*sargassaceae*) biomass. For this biomass, 230 kt/yr of xylitol and 116 kt/yr of sorbitol are obtained, reaching an environmental factor of 0.141 kg CO_2_/kg biomass with an investment of up to 120.5 M€ for a production cost of 0.25 €/kg. Further validation of the process at the pilot plant is necessary before actual industrial production using the concepts presented in this work.

## Supporting Information Available

Data on the development of models and fittings; results on the break down of costs for different biomasses; operating variables for AFEX pretreatment and dilute acid pretreatment; xylitol and sorbitol production by the fermentation pathway (PDF)

## Nomenclature

*a*, *b*, *c*: fitting parameters for ammonia recovery column operations
*d*, *e*: fitting parameters for xylitol conversion
*d*_1_, *d*_2_, *d*_3_: fitting coefficients to obtain *d*
*e*_1_, *e*_2_, *e*_3_: fitting coefficients to obtain *e*
ammonia_ratio: ratio of ammonia added vs dry biomass to AFEX pretreatment (g/g)
conc_acid_mix: acid concentration at pretreatment in weight percentage
*C_i_*: material cost ($/g or $/W)
*C*_p*_i_*_: heat capacity of component *i* (kJ/(kg·°C))
*D*_*i*,*k*_: flow of component *i* in distillate of column *k*
*D*_t_: temperature increment
enzyme_add: ratio of enzyme added to hydrolysis for acid pretreatment as a function of the glucan (g/g)
*E*: environmental index (kg CO_2_/kg biomass)
*EF*_H_2__: environmental factor for hydrogen (kg CO_2_/kg H_2_)
*EF*_steam_: environmental factor for steam (kg CO_2_/kg steam)
*c*_r_*i*__: flow of sugar *i* in the crystal stream (kg/s)
*fc*_H_2__: flow of consumed hydrogen (kg/s)
*fc*_ sorbitol_: flow of purified sorbitol (kg/s)
*fc*_ xylitol_: flow of purified xylitol (kg/s)
*f*_d_*i*__: flow of sugar *i* in the evaporator feed (kg/s)
*l*_*i*_: flow of sugar *i* in the solution flow (kg/s)
*F*_d_: feed to multieffect column (kg/s)
Δ*H*: reaction enthalpy (kJ/mol)
Δ*H*_form *i*_: formation enthalpy of component *i* at 25 °C (kJ/kg)
*H*_cr_: crystal enthalpy (kW)
*H*_E_: vapor enthalpy (kW)
*H*_e_: condensed vapor enthalpy (kW)
*H*_F_d__: feed enthalpy (kW)
*H*_L_: solution enthalpy (kW)
*H*_S_: steam enthalpy (kW)
*H*_s_: condensated steam enthalpy (kW)
LoadAmmonia_water: mass ratio between ammonia and water
*m*_(*J*, unit, unit 1)_: mass flow of component *J* from unit to unit 1 (kg/s)
*m*_*i*_: molality of sugar *i* (mol/kg)
*K*_b_: ebullioscopy water constant (0.512 kg/(mol·K))
*K*_isomerization_: isomerization glucose constant
*P*: pressure (bar)
*P_i_*: prices of component *i* (€/kg-€/kWh)
*P_j_*: operating pressure of evaporator *j* (bar)
*P_k_*: pressure of column *k*
*Q*_(unit)_: thermal energy involved in unit (W)
*Q*_b*_k_*_: thermal flow in boiler of column *k*
*Q*_w*_k_*_: thermal flow in condenser of column *k*
*R*: reflux ratio
ReactionTime: reaction time in catalytic reactors CR-1 and CR-2
*T*_(Unit, Unit 1)_: temperature of the stream from unit to unit 1 (°C)
*T*_b*_k_*_: temperature in the boiler of column *k*
*T*_c*_k_*_: temperature in the condenser of column *k*
time_pret (min): time for acid pretreatment
*T*: operating temperature (°C)
*T*_acid: operating temperature in acid pretreatment (°C)
*T*_afex: operating temperature in AFEX pretreatment (°C)
*T*_Cr_: operating crystal temperature (°C)
*T*_eb_: boiling temperature (°C)
*T*_E_: vapor overhead temperature (°C)
*T*_F_d__: operating feed temperature (°C)
*T*_L_: operating solution temperature (°C)
*T*_ref_: operating reference temperature (25 °C)
*T*_S_: operating steam temperature (°C)
*T_j_*: operating temperature in evaporator *j* (°C)
*T*_*j*+1_: operating temperature in evaporator *j* + 1 (°C)
time_pret: time for AFEX pretreatment (min)
water_pret: ratio of water added to AFEX pretreatment function of the dry biomass (g/g)
*W*_*i*,*k*_: flow of component *i* in the residue of column *k*
*W*_(unit)_: electrical power involved in the unit (W)
*x*_*i*_: sugar *i* solubility (g/100 water-kg/kg water)
*X*_*i*_: sugar *i* conversion
yield: yield of the pretreatment/unit
**Symbols**
λ: latent heat steam (kJ/kg)
η: separation ratio in the column
[]: concentration (mol/L-g/100 mL water-kg/kg water)
Wa: water
CO_2_: carbon dioxide
CH_1.8_O_0.5_N_0.2_: cells
C_5_H_10_O_5_: xylose
C_5_H_12_O_5_: xylitol
C_6_H_12_O_6_: fructose–glucose
C_6_H_14_O_6_: sorbitol
CaO: lime
CaSO_4_: gypsum
H_2_: hydrogen
H_2_SO_4_: sulfuric acid
NH_3_: ammonia
O_2_: oxygen

## References

[ref1] Aden A.; Foust T. Technoeconomic analysis of the dilute sulfuric acid and enzymatic hydrolysis process for the conversion of corn stover to ethanol. Cellulose 2009, 16, 535–545. 10.1007/s10570-009-9327-8.

[ref2] Keshwani D. R.; Cheng J. J. Switchgrass for bioethanol and other value-added applications: A review. Bioresour. Technol. 2009, 100, 1515–1523. 10.1016/j.biortech.2008.09.035. 18976902

[ref3] Martín M.; Grossmann I. E. Energy Optimization of Bioethanol Production via Gasification of Switchgrass. AIChE J. 2011, 57, 3408–3428. 10.1002/aic.12544.

[ref4] Karuppiah R.; Peschel A.; Grossmann I. E.; Martín M.; Martinson W.; Zullo L. Energy optimization for the design of corn-based ethanol plants. AIChE J. 2008, 54, 1499–1525. 10.1002/aic.11480.

[ref5] Kazi F. K.; Patel A. D.; Serrano-Ruiz J. C.; Dumesic J. A.; Anexa R. P. Techno-economic analysis of dimethylfuran (DMF) and hydroxymethylfurfural(HMF) production from pure fructose in catalytic processes. Chem. Eng. J. 2011, 169, 329–338. 10.1016/j.cej.2011.03.018.

[ref6] Yemis O.; Mazza G. Optimization of furfural and 5-hydroxymethylfurfural production from wheat straw by a microwave-assisted process. Bioresour. Technol. 2012, 109, 215–223. 10.1016/j.biortech.2012.01.031. 22297050

[ref7] Marlière P. Production of Alkenes by Enzymatic Decarboxylation of 3-Hydroxyalkanoic Acids. U.S Patent US20110165644A12011.

[ref8] Ribeiro L.; Delgado J. J.; Orfao J.; Pereira M. F. A one-pot method for the enhanced production of xylitol directly from hemicellulose (corncob xylan). RSC Adv. 2016, 6, 95320–95327. 10.1039/C6RA19666G.

[ref9] Marques C.; Tarek R.; Sara M.; Brar S. K. Sorbitol production from biomass and its global market. In Platform Chemical Biorefinery; Elsevier, 2016; Vol. 12, pp 217–227.

[ref10] Holladay J.; Bozell J.; White J.; Johnson D. Top Value-Added Chemicals from Biomass: Volume II-Results of Screening for Potential Candidates from Biorefinery Lignin, DOE Report PNNL 16983; Pacific Northwest National Lab: Richland, WA, 2007.

[ref11] Werpy T.; Petersen G.; Aden A.; Bozell J.; Holladay J.; Manheim A.; Eliot D.; Lasure L.; Jones S. Top Value-Added Chemicals from Biomass: Volume I- Results of Screening for Potential Candidates from Sugars and Synthesis Gas, DOE/GO-102004-1992; National Renewable Energy Lab: Golden, CO, 2004.

[ref12] Taylor R.; Nattrass L.; Alberts G. From the Sugar Platform to Biofuels and Biochemicals: Final Report for the European Commission Directorate-General Energy, No ENER/C2/423-2012/SI2.673791. V2.1, 2015.

[ref13] Gérardy R.; Debecker D. P.; Estager J.; Luis P.; Monbaliu J. C. M. Continuous Flow Upgrading of Selected C2-C6 Platform Chemicals Derived from Biomass. Chem. Rev. 2020, 120, 7219–7347. 10.1021/acs.chemrev.9b00846. 32667196

[ref14] Dusselier M.; Mascal M.; Sels B. F. Top Chemical Opportunities from Carbohydrate Biomass: A Chemist’s View of the Biorefinery. Top. Curr. Chem. 2014, 355, 1–40. 10.1007/128_2014_544 24842622

[ref15] Ribeiro L. S.; Melo Orfao J. J.; de Pereisa M. F. R. Direct catalytic production of sorbitol from waste cellulosic materials. Bioresour. Technol. 2017, 232, 152–158. 10.1016/j.biortech.2017.02.008. 28222384

[ref16] Van Gorp K.; Boerman E.; Cavenaghi C. V.; Berben P. H. Catalytic hydrogenation of fine chemicals: sorbitol production. Catal. Today 1999, 52, 349–361. 10.1016/S0920-5861(99)00087-5.

[ref17] Chun U. H.; Rogers P. L. The Simultaneous Production of Sorbitol from Fructose and Gluconic Acid from Glucose using an oxidoreductase of *Zymomonas mobilis*. Appl. Microbiol. Biotechnol. 1988, 29, 19–24. 10.1007/BF00258345.

[ref18] Silveira M. M.; Jonas R. The biotechnological production of sorbitol. Appl. Microbiol. Biotechnol. 2002, 59, 400–408. 10.1007/s00253-002-1046-0. 12172602

[ref19] Illanes A.; Zuñiga M. E.; Contreras S.; Guerrero A. Reactor design for the enzymatic isomerization of glucose to fructose. Bioprocess Eng. 1992, 7, 199–204. 10.1007/BF00369546.

[ref20] Takasaki Y. Kinetic and Equilibrium Studies on d-Glucose-dFructose Isomerization Catalyzed by Glucose Isomerase from *Streptomyces* sp. Agric. Biol. Chem. 1967, 31, 309–313. 10.1080/00021369.1967.10858809.

[ref21] Kapandji K. K.; Haigh K. F.; Görgens J. F. Techno-Economic Analysis of Chemically Catalysed Lignocellulose Biorefineries at A Typical Sugar Mill: Sorbitol or Glucaric Acid and Electricity Co-Production. Bioresour. Technol. 2019, 289, 121635 10.1016/j.biortech.2019.121635. 31254898

[ref22] Srivani K.; Pydi Setty Y. Parametric optimization of xylitol production from xylose by fermentation. Asia-Pac. J. Chem. Eng. 2012, 7, 280–284. 10.1002/apj.1645.

[ref23] Rafiqul S. M.; Mimi Sakinah A. M. Processes for the Production of Xylitol—A Review. Food Rev. Int. 2013, 29, 127–156. 10.1080/87559129.2012.714434.

[ref24] Mountraki A. D.; Koutsospyros K. R.; Mlayah B. B.; Kokossis A. C. Selection of Biorefinery Routes: The Case of Xylitol and its Integration with an Organosolv Process. Waste Biomass Valorization 2017, 8, 2283–2300. 10.1007/s12649-016-9814-8.

[ref25] Ozudogru R.; Nieder-Heitmann M.; Haigh K. F.; Görgens J. F. Techno-economic analysis of product biorefineries utilizing sugarcane lignocelluloses: Xylitol, citric acid and glutamic acid scenarios annexed to sugar mills with electricity co-production. Ind. Crops Prod. 2019, 133, 259–268. 10.1016/j.indcrop.2019.03.015.

[ref26] Alvira P.; Tomás-Pejó E.; Ballesteros M.; Negro M. J. Pretreatment technologies for an efficient bioethanol production process based on enzymatic hydrolysis: A review. Bioresour. Technol. 2010, 101, 4851–4861. 10.1016/j.biortech.2009.11.093. 20042329

[ref27] Sun Y.; Cheng J. Hydrolysis of lignocellulosic materials for ethanol production: a review. Bioresour. Technol. 2002, 83, 1–11. 10.1016/S0960-8524(01)00212-7. 12058826

[ref28] Taherzadeh M.; Karimi K. Pretreatment of Lignocellulosic Wastes to improve ethanol and biogas production: A review. Int. J. Mol. Sci. 2008, 9, 1621–1651. 10.3390/ijms9091621. 19325822 PMC2635757

[ref29] Kazi F. K.; Fortman J. A.; Anex R. P.; Hsu D. D.; Aden A.; Dutta A.; Kothandaraman G. Technoeconomic comparison of process technologies for biochemical ethanol production from corn stover. Fuel 2010, 89, 20–28. 10.1016/j.fuel.2010.01.001.

[ref30] Piccolo C.; Bezzo F. A techno-economic comparison between two technologies for bioethanol production from lignocelluloses. Biomass Bioenergy 2009, 33, 478–491. 10.1016/j.biombioe.2008.08.008.

[ref31] Zhang J.; Li J. B.; Wu S. B.; Liu Y. Advances in the Catalytic Production and Utilization of Sorbitol. Ind. Eng. Chem. Res. 2013, 52, 11799–11815. 10.1021/ie4011854.

[ref32] Alizadeh H.; Teymouri F.; Gilbert T. I.; Dale B. E. Pretreatment of switchgrass by ammonia fiber explosion (AFEX). Appl. Biochem. Biotechnol. 2005, 124, 1133–1141. 10.1385/ABAB:124:1-3:1133. 15930586

[ref33] Murnen H. K.; Balan V.; Chundawat S. P. S.; Bals B.; Sousa L.; da C.; Dale B. E. Optimization of Ammonia fiber expansion (AFEX) pretreatment and enzymatic hydrolysis of Miscanthus x giganteus to Fermentable sugars. Biotechnol. Prog. 2007, 23, 846–850. 10.1002/bp070098m. 17585779

[ref34] Aristizábal V.; Gómez Á. Biorefineries based on coffee cut-stems and sugarcane bagasse: Furan-based compounds and alkanes as interesting products. Bioresour. Technol. 2015, 196, 480–489. 10.1016/j.biortech.2015.07.057. 26280100

[ref35] Gregg D.; Saddler J. N. Bioconversion of lignocellulosic residue to ethanol: Process flowsheet development. Biomass Bioenergy 1995, 9, 287–302. 10.1016/0961-9534(95)00097-6.

[ref36] Hamelinck C. N.; Hooijdonk G. V.; Faaij A. P. C. Ethanol from lignocellulosic biomass: techno-economic performance in short-, middle- and long-term. Biomass Bioenergy 2005, 28, 384–410. 10.1016/j.biombioe.2004.09.002.

[ref37] Wooley R.; Ruth M.; Sheehan J.; Ibsen K.; Majdeski H.; Galvez A. In Lignocellulosic Biomass to Ethanol Process Design and Economics Utilizing Co-current Dilute Acid Prehydrolysis and Enzymatic Hydrolysis Current and Futuristic Scenarios, No. NREL/TP-580-26157; National Renewable Energy Laboratory: Golden, CO, 1999; p 132.

[ref38] Silva S. S.; Roberto I. C.; Felipe M. G.; Mancilha I. M. Batch fermentation of xylose for xylitol production in stirred tank bioreactor. Process Biochem. 1996, 31, 549–553. 10.1016/S0032-9592(96)00002-7.

[ref39] Pham T. N.; Samikannu A.; Rautio A. R.; Juhasz K. L.; Konya Z.; Wärna J.; Kordas K.; Mikkola J. P. Catalytic hydrogenation od D-xylose over Ru decorated carbon foam catalyst in a SpinChem rotating bed reactor. Top. Catal. 2016, 59, 1165–1177. 10.1007/s11244-016-0637-4.

[ref40] Gallezot P.; Nicolaus N.; Fleche G.; Fuertes P.; Perrard A. Glucose hydrogenation on ruthenium catalysts in a trickle-bed reactor. J. Catal. 1998, 180, 51–55. 10.1006/jcat.1998.2261.

[ref41] Hernández-Mejıa C.; Raja E.; Olivos-Suárez A.; Gascón J.; Greer H. F.; Zhou W.; Rothenberg G.; Shiju R. N. Ru/TiO2-catalysed hydrogenation of xylose: the role of crystal structure of the support. Catal. Sci. Technol. 2015, 6, 577–582. 10.1039/C5CY01005E.

[ref42] Luo C.; Wang S.; Liu H. Cellulose conversion into polyols catalyzed by reversibly formed acids and supported ruthenium clusters in hot water. Angew. Chem., Int. Ed. 2007, 46, 7636–7639. 10.1002/anie.200702661. 17763479

[ref43] Broekhuis R. R.; Budhlall B. M.; Nordquist A. F. Monolith Catalytic Process for Producing Sorbitol: Catalyst Development and Evaluation. Ind. Eng. Chem. Res. 2004, 43, 5146–5155. 10.1021/ie0400393.

[ref44] Kusserow B.; Schimpf S.; Claus P. Hydrogenation of Glucose to Sorbitol over Nickel and Ruthenium Catalysts. Adv. Synth. Catal. 2003, 345, 1–323. 10.1002/adsc.200390024.

[ref45] Martín M. Industrial Chemical Process: Analysis and Design; Elsevier: Oxford, U.K., 2016.

[ref46] Mani S.; Tabil L. G.; Sokhansanj S. Grinding performance and physical properties of wheat and barley straws, corn stover and switchgrass. Biomass Bioenergy 2004, 27, 339–352. 10.1016/j.biombioe.2004.03.007.

[ref47] Kumar P.; Barrett D. M.; Delwiche M. J.; Stroeve P. Methods for pretreatment of lignocellulosic biomass for efficient hydrolysis and biofuel production. Ind. Eng. Chem. Res. 2009, 48, 3713–3729. 10.1021/ie801542g.

[ref48] Mosier N.; Wyman C.; Dale B.; Elander R.; Lee Y. Y.; Holtapple M.; Ladish M. Features of promising technologies for pretreatment of lignocellulosic biomass. Bioresour. Technol. 2005, 96, 673–686. 10.1016/j.biortech.2004.06.025. 15588770

[ref49] Sierra R.; Smith A.; Granda C.; Holtzapple M. T. Producing Fuels and Chemicals from lignocellulosic Biomass. Chem. Eng. Prog. 2008, 10–18.

[ref50] Tao L.; Aden A.; Elander R. T.; Pallapolu V. R.; Lee Y. Y.; Garlock R. J. Process and technoeconomic analysis of leading pretreatment technologies for lignocellulosic ethanol production using switchgrass. Bioresour. Technol. 2011, 102, 11105–11114. 10.1016/j.biortech.2011.07.051. 21865030

[ref51] Garlock R. J.; Balan V.; Dale B. E. Optimization of AFEX pretreatment conditions and enzyme mixtures to maximize sugar release from upland and lowland switchgrass. Bioresour. Technol. 2012, 104, 757–768. 10.1016/j.biortech.2011.11.034. 22138594

[ref52] Holtzapple M. T.; Jun J.-A.; Ashok G.; Patibandla S. L.; Dale B. E. The Ammonia Freeze Explosion (AFEX) Process: A Practical Lignocellulose Pretreatment. Appl. Biochem. Biotechnol. 1992, 28/29, 59–74. 10.1007/BF02922589.

[ref53] Sendich E. N.; Lase M.; Kim S.; Alizadeh H.; Laureano-Perez L.; Dale B.; Lynd L. Recent process improvements for the ammonia fiber expansion (AFEX) process and resulting reductions in minimum ethanol selling price. Bioresour. Technol. 2008, 99, 8429–8435. 10.1016/j.biortech.2008.02.059. 18440810

[ref54] Galán G.; Martin M.; Grossmann I. E. Integrated renewable production of ETBE from Switchgrass. ACS Sustainable Chem. Eng. 2019, 7, 8943–8953. 10.1021/acssuschemeng.9b01004. PMC8592025 34795467

[ref55] Canettieri E. V.; Rocha G. J. M.; Carvalho J. A.; De Almeida e Silva J. B. Optimization of acid hydrolysis from the hemicellulosic fraction of Eucalyptus grandis residue using response surface methodology. Bioresour. Technol. 2007, 98, 422–428. 10.1016/j.biortech.2005.12.012. 16473004

[ref56] Schell D. J.; Ruth M. F.; Tucker M. P. Modeling the Enzymatic Hydrolysis of Dilute-Acid Pretreated Douglas Fir. Appl. Biochem. Biotechnol. 1999, 77, 67–81. 10.1385/ABAB:77:1-3:67.

[ref57] Shi J.; Ebrik M. A.; Wyman C. E. Sugar yields from dilute sulfuric acid and sulfur dioxide pretreatments and subsequent enzymatic hydrolysis of switchgrass. Bioresour. Technol. 2011, 102, 8930–8938. 10.1016/j.biortech.2011.07.042. 21835614

[ref58] Lavarack B. P.; Griffin G. J.; Rodman D. The acid hydrolysis of sugarcane bagasse hemicellulose to produce xylose, arabinose, glucose and other products. Biomass Bioenergy 2002, 23, 367–380. 10.1016/S0961-9534(02)00066-1.

[ref59] Schell D. J.; Farmer J.; Newman M.; McMillan J. D. Dilute Sulfuric Acid Pretreatment of Corn Stover in Pilot—Scale reactor. Appl. Biochem. Biotechnol. 2003, 105/108, 69–85. 10.1385/ABAB:105:1-3:69. 12721476

[ref60] Zhang S.; Marechal F.; Gassner M.; Perin-Levasseur Z.; Qi W.; Ren Z.; Yan Y.; Favrat D. Process Modeling and Integration of Fuel Ethanol Production from Lignocellulosic Biomass Based on Double Acid Hydrolysis. Energy Fuels 2009, 23, 1759–1765. 10.1021/ef801027x.

[ref61] National Lime Association. Using Lime for Acid Neutralization. A Proven Solution!, 2012. http://www.lime.org.

[ref62] Dasgupta D.; Bandhu S.; Adhikari D. K.; Ghosh D. Challenges and prospects of xylitol production with whole cell. Microbiol. Res. 2017, 197, 9–21. 10.1016/j.micres.2016.12.012. 28219529

[ref63] Yadav M.; Mishra D. K.; Hwang J.-S. Catalytic hydrogenation of xylose to xylitol using ruthenium catalyst on NiO modified TiO 2 support. Appl. Catal., A 2012, 425–426, 110–116. 10.1016/j.apcata.2012.03.007.

[ref64] Mikkola J.-P.; Vainio H.; Salmi T.; Sjoholm R.; Ollonqvist T.; Vayrynen J. Deactivation kinetics of Mo-supported Raney Ni catalyst in the hydrogenation of xylose to xylitol. Appl. Catal., A 2000, 196, 143–155. 10.1016/S0926-860X(99)00453-6.

[ref65] Romero A.; Alonso E.; Sastre A.; Nieto-Márquez A. Conversion of biomass into sorbitol: Cellulose hydrolysis on MCM-48 and D-Glucose hydrogenation on Ru/MCM-48. Microporous Mesoporous Mater. 2016, 224, 1–8. 10.1016/j.micromeso.2015.11.013.

[ref66] Manteca P.; Martin M. Integrated facility for power plant waste processing. Ind. Eng. Chem.Res. 2019, 58, 6155–6152. 10.1021/acs.iecr.8b04029.

[ref67] Yee T. F.; Grossmann I. E.; Kravanja Z. Simultaneous optimization models for heat integration—I. Area and energy targeting and modeling of multi-stream exchangers. Comput. Chem. Eng. 1990, 14, 1154–1164. 10.1016/0098-1354(90)85009-Y.

[ref68] Sinnott R. K. Coulson and Richardson’s Chemical Engineering. In Chemical Engineering Design; Butterworth-Heinemann: Oxford, 1999; Vol. 6.

[ref69] Pérez-Uresti S.; Martin M.; Jiménez-Gutiérrez A. Estimation of renewable-based steam costs. Appl. Energy 2019, 250, 1120–1131. 10.1016/j.apenergy.2019.04.189.

[ref70] Almena A.; Martín M. Techno-economic analysis of the production of epiclorhidrin from glycerol. Ind. Eng. Chem. Res. 2016, 55, 3226–3238. 10.1021/acs.iecr.5b02555.

[ref71] Martín M.; Grossmann I. E. Optimization simultaneous production of ethanol and i-butene from Switchgrass. Biomass Bioenergy 2014, 61, 93–103. 10.1016/j.biombioe.2013.11.022.

[ref72] Valente A.; Iribarren D.; Dufour J. Prospective carbon footprint comparison of hydrogen options. Sci. Total Environ. 2020, 728, 138212 10.1016/j.scitotenv.2020.138212. 32361105

[ref73] U.S. Environmental Protection Agency (EPA). Emissions Factors for greenhouse Gas Inventories; Center For Corporate Climate Leadership, 2018.

[ref74] Rosales-Calderón Ó.; Arantes V. A review on commercial-scale high-value products that can be produced alongside cellulosic ethanol. Biotechnol. Biofuels 2019, 12, 240 10.1186/s13068-019-1529-1. 31624502 PMC6781352

