# Supplementary material for: Integrated Renewable Production of Sorbitol and Xylitol from Switchgrass
Source: Ind Eng Chem Res. 2021 Apr 12;60(15):5558–73. doi: 10.1021/acs.iecr.1c00397 (PMC8592025; doi:10.1021/acs.iecr.1c00397)
Supplement: Supplementary file 1 — ie1c00397_si_001.pdf [file ie1c00397_si_001.pdf]

Integrated Renewable Production of Sorbitol and Xylitol from Switchgrass

Guillermo Galán[a], Mariano Martín[a*], Ignacio E. Grossmann[b].

[a]*Department of Chemical Engineering. University of Salamanca. Plz Caidos 1-5, 37008, Salamanca, Spain*

[b]*Department of Chemical Engineering. Carnegie Mellon University, 5000 Forbes Ave, 15213, Pittsburgh PA, USA*

mraiano.m3@usal

Supplementary Material

AFEX pretreatment

Table S1.- Range of operating variables for dilute acid pretreatment

	Lower bound	Upper bound
T (°C)	90	180
Ammonia added ($g \cdot g^{-1}$)	0.5	2
Water added ($g \cdot g^{-1}$)	0.5	2
Residence time (min)	5	30

$$\text{Yield} = 0.01\cdot(-88.7919 + 26.5272\cdot \text{amonia\_ratio} - 13.6733\cdot \text{water\_pret} + 1.6561\cdot \text{T\_afex} + 3.6793\cdot \text{time\_pret} - 4.4631\cdot \text{amonia\_ratio}^2 - 0.0057\cdot \text{T\_afex}^2 - 0.0270\cdot \text{time\_pret}^2 - 0.4064\cdot \text{amonia\_ratio}\cdot \text{time\_pret} + 0.1239\cdot \text{water\_pret}\cdot \text{T\_afex} - 0.0132\cdot \text{T\_afex}\cdot \text{time\_pret});$$

$$Q_{(HX2)} = \sum_j m_{(J,HX2,Col1)} \cdot c_{p,j} \cdot \left(T_{(HX2,Col1)} - T_{(Valv1,HX2)}\right)$$

Where:

$$T_{(\mathrm{HX2,Col1})} = a_{(NH_3/Water)} \cdot P^2 + b_{(NH_3/Water)} \cdot P + c_{(NH_3/Water)}$$

$$a_{(NH_3/Water)} = 1.0152 \cdot \left(\mathrm{Load}_{\mathrm{Ammonia\_Water}}\right)^2 - 0.3996 \cdot \left(\mathrm{Load}_{\mathrm{Ammonia\_Water}}\right) - 2.118$$

$$b_{(NH_3/Water)} = -11.344\left(\mathrm{Load}_{\mathrm{Ammonia\_Water}}\right)^2 + 8.6088 \cdot \left(\mathrm{Load}_{\mathrm{Ammonia\_Water}}\right) + 25.159;$$

$$c_{(NH_3/Water)} = 68.912\left(\mathrm{Load}_{\mathrm{Ammonia\_Water}}\right)^2 - 174.79 \cdot \left(\mathrm{Load}_{\mathrm{Ammonia\_Water}}\right) + 59.214$$

And the purity

$$Purity = a_{(NH_3/Water)} \cdot P^2 + b_{(NH_3/Water)} \cdot P + c_{(NH_3/Water)}$$

$$a_{(NH_3/Water)} = -0.5792 \cdot \left(\mathrm{Load}_{\mathrm{Ammonia\_Water}}\right)^2 - 0.9987 \cdot \left(\mathrm{Load}_{\mathrm{Ammonia\_Water}}\right) + 1.48$$

$$b_{(NH_3/Water)} = 9.9096\left(\mathrm{Load}_{\mathrm{Ammonia\_Water}}\right)^2 - 0.3672 \cdot \left(\mathrm{Load}_{\mathrm{Ammonia\_Water}}\right) - 12.319$$

$$c_{(NH_3/Water)} = -81.468\left(\mathrm{Load}_{\mathrm{Ammonia\_Water}}\right)^2 + 127.72\left(\mathrm{Load}_{\mathrm{Ammonia\_Water}}\right) + 56.404$$

Purity<99;

Purity>80

And the recovery yield of ammonia is given by eq. (5)

$$Yield = -0.1123 \cdot \left(\mathrm{Load}_{\mathrm{\_Ammonia\_Water}}\right)^2 + 0.2055 \cdot \left(\mathrm{Load}_{\mathrm{\_Ammonia\_Water}}\right) + 99.889;$$

Rend<99.99;

Rend>99.9;

The energy balance to the column is modeled using the following surrogate models for the condenser and the reboiler respectively (kW) per:

$$Q_{\mathrm{condenser}} = \left(4.8709 \cdot \left(\mathrm{Load}_{\mathrm{\_Ammonia\_Water}}\right) + 1.4989\right) \frac{f_{c(water)}[kg/s]}{0.028}$$

$$Q_{\mathrm{Re}boiler} = \left(9.4089 \cdot \left(\mathrm{Load}_{\mathrm{\_Ammonia\_Water}}\right) + 2.7523\right) \frac{f_{c(water)}[kg/s]}{0.028}$$

The exit temperatures of the column are computed as follows:

$$T_{(Col1,Mix2)} = a_{(NH_3/Water)} \cdot P^2 + b_{(NH_3/Water)} \cdot P + c_{(NH_3/Water)}$$

$$a_{(NH_3/Water)} = -15.315 \cdot \left(\mathrm{Load}_{\mathrm{Ammonia\_Water}}\right)^2 + 13.426 \cdot \left(\mathrm{Load}_{\mathrm{Ammonia\_Water}}\right) - 4.9178$$

$$b_{(NH_3/Water)} = 127.73\left(\mathrm{Load}_{\mathrm{Ammonia\_Water}}\right)^2 - 103.7 \cdot \left(\mathrm{Load}_{\mathrm{Ammonia\_Water}}\right) + 47.523$$

$$c_{(NH_3/Water)} = -238.48\left(\mathrm{Load}_{\mathrm{Ammonia\_Water}}\right)^2 + 116.32\left(\mathrm{Load}_{\mathrm{Ammonia\_Water}}\right) + 25.337$$

$$\mathrm{T}_{(\mathrm{Col1,Mix3})} = -1.8617 \cdot P^2 + 24.16 \cdot P + 77.491$$

Dilute Acid pretreatment

Table S2.- Range of operating variables for dilute acid pretreatment

	Lower bound	Upper bound
T (ºC)	140	180
Acid concentration ($g \cdot g^{-1}$)	0.005	0.02
Residence time (min)	1	80
Enzyme load ($g \cdot g^{-1}$)	0.0048	0.0966

The yield of glucose is given by:

$$\begin{aligned}
&\mathrm{yield_cellu} = -0.00055171 + 0.00355819 \cdot \mathrm{T_acid} + 0.00067402 \cdot \mathrm{conc_acid_mix} + \\
&\mathrm{time_pret} \cdot 0.00100531 - \mathrm{enzyme_add} \cdot 0.0394809 - 0.0186704 \cdot \mathrm{T_acid} \cdot \mathrm{conc_acid_mix} \\
&+ 0.00043556 \cdot \mathrm{T_acid} \cdot \mathrm{time_pret} + 0.0002265 \cdot \mathrm{T_acid} \cdot \mathrm{enzyme_add} \\
&- 0.0013224 \cdot \mathrm{conc_acid_mix} \cdot \mathrm{time_pret} - 0.00083728 \cdot \mathrm{time_pret} \cdot \mathrm{enzyme_add} \\
&+ 0.044353 \cdot \mathrm{conc_acid_mix} \cdot \mathrm{enzyme_add} + 0.000014412 \cdot \mathrm{T_acid}^2;
\end{aligned}$$

The yield of xylose is given by:

$$yield\_hemi = -0.00015791 - 0.00056353 \cdot T\_acid + 0.000694361 \cdot conc\_acid\_mix$$
$$-0.00014507 \cdot time\_pret - enzyme\_add \cdot 0.01059248 - 0.02142606 \cdot T\_acid \cdot conc\_acid\_mix$$
$$+0.000694055 \cdot T\_acid \cdot time\_pret + 0.00013559 \cdot T\_acid \cdot enzyme\_add$$
$$-0.00145712 \cdot conc\_acid\_mix \cdot time\_pret + 0.04769633 \cdot conc\_acid\_mix \cdot enzyme\_add$$
$$-0.00138362 \cdot time\_pret \cdot enzyme\_add + 0.0000059419 \cdot T\_acid^2$$

3.2. Xylitol and Sorbitol Production by fermentation pathway

the best concentration. Figure 1 shows the representation of the fructose conversion data achieved by *Zymomonas mobilis* as a function of its concentration[13]. In this step the traces of glucose convert to gluconic acid are neglected. We assume the traces of glucose can be used as a nutrient by the bacteria.

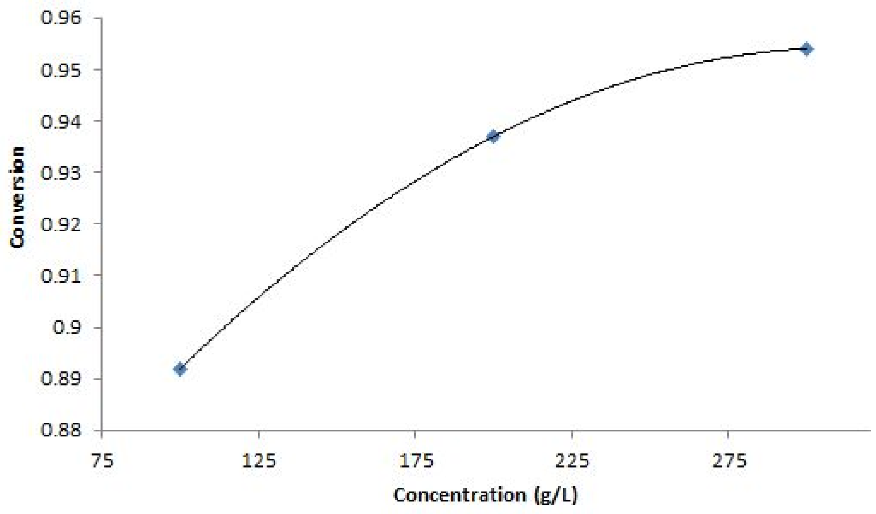

Figure S1. Fructose conversion with the concentration

Solubilities

Xylose solubility

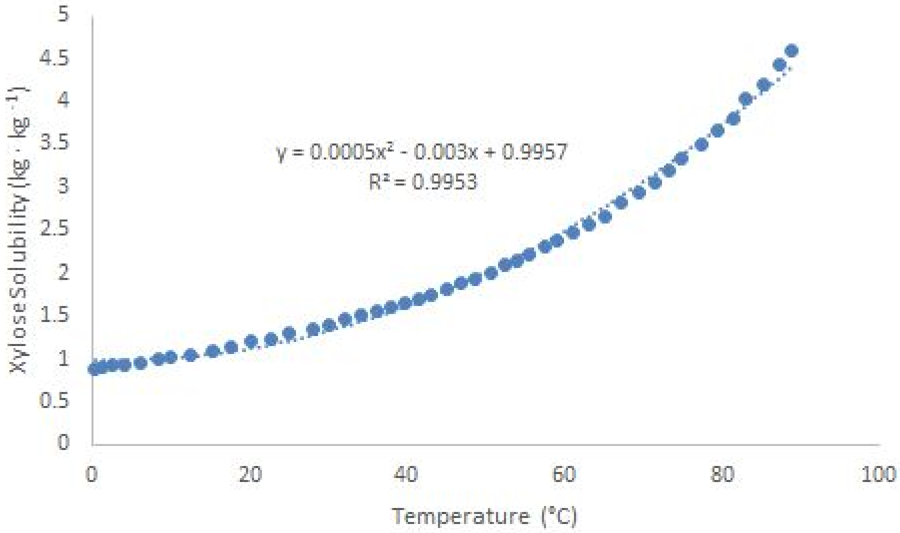

Figure S2. Xylose solubility

Xylitol solubility

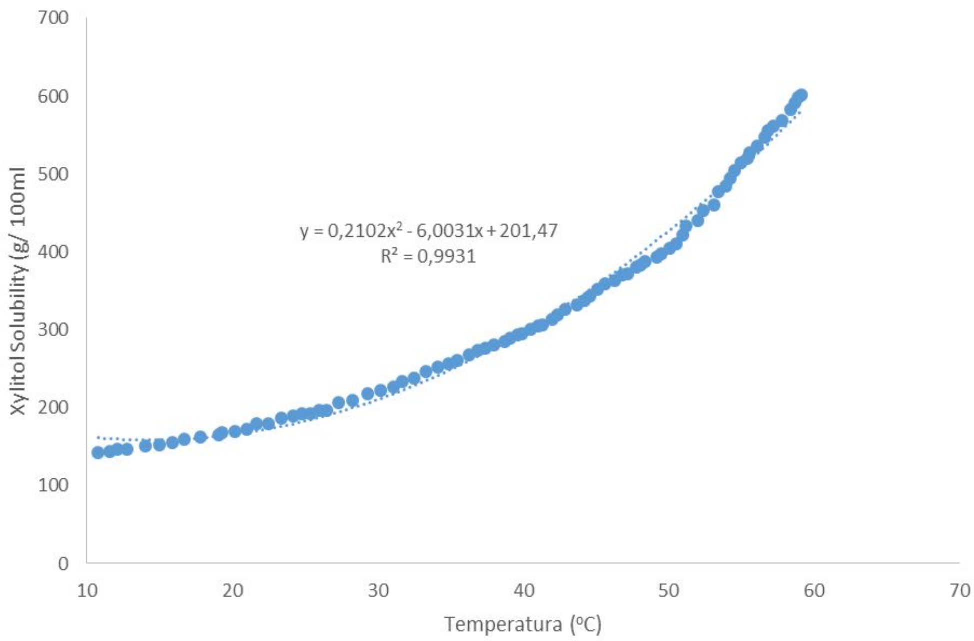

Figure S3. Xylitol solubility

Fructose solubility

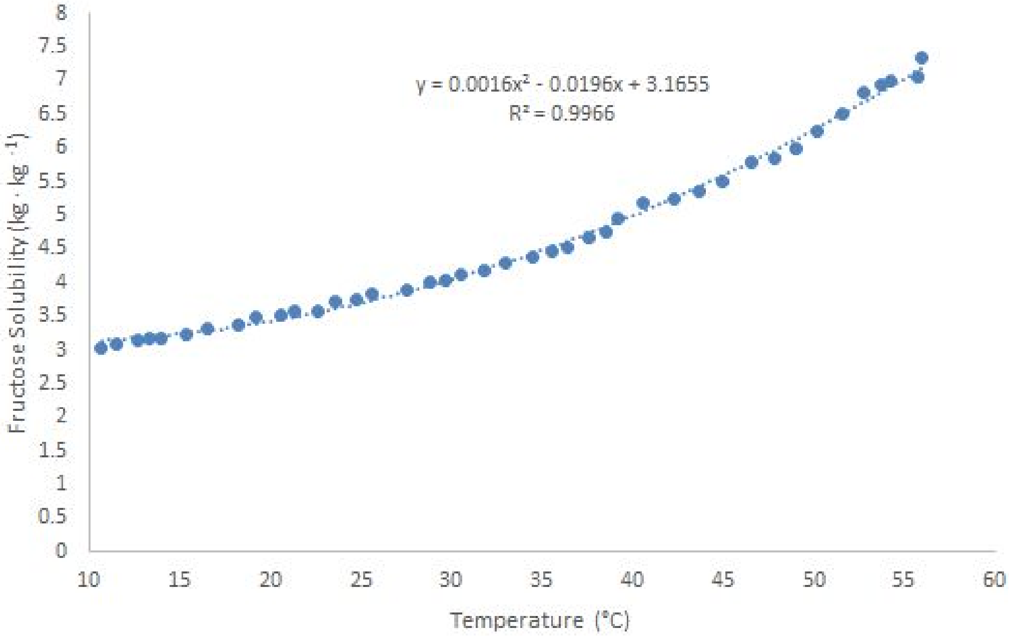

Figure S4. Fructose solubility

Sorbitol solubility

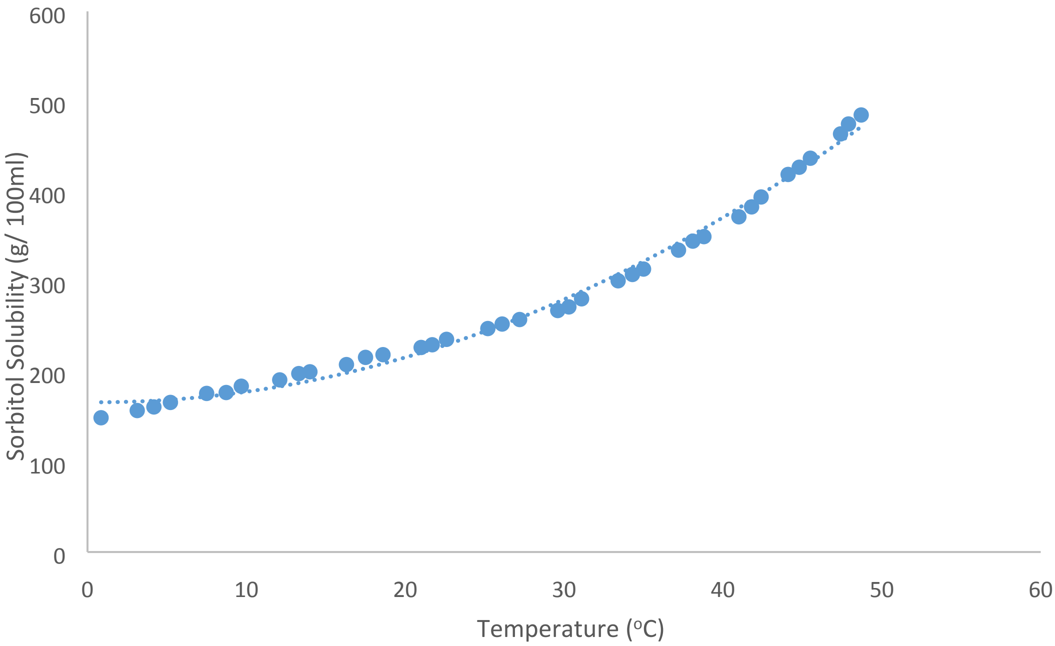

Figure S5. Sorbitol solubility

Xylitol catalysis model

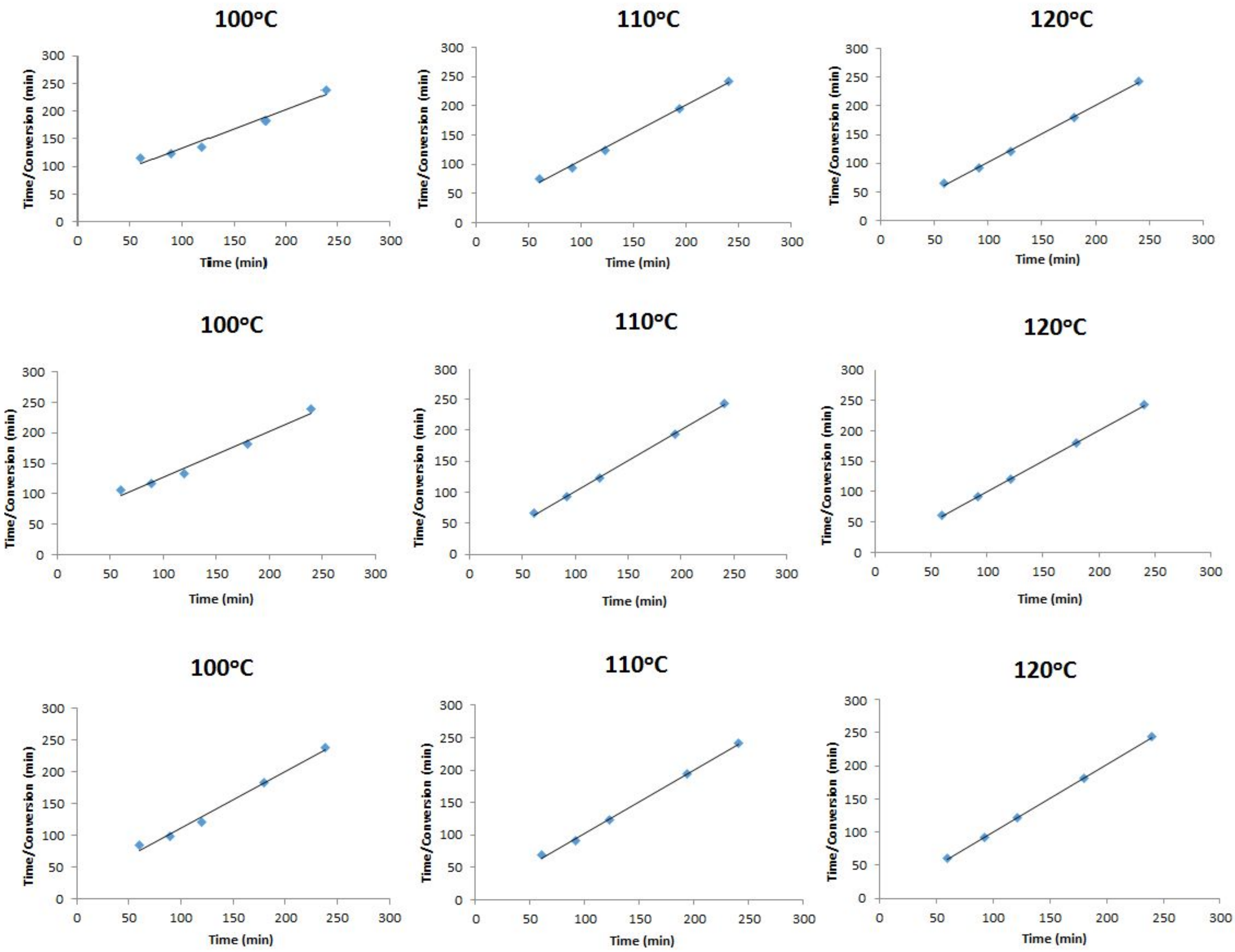

Figure S6. Linearization for 40, 50 and 60 bar

Parameters fitting

Parameter d for 40 bar

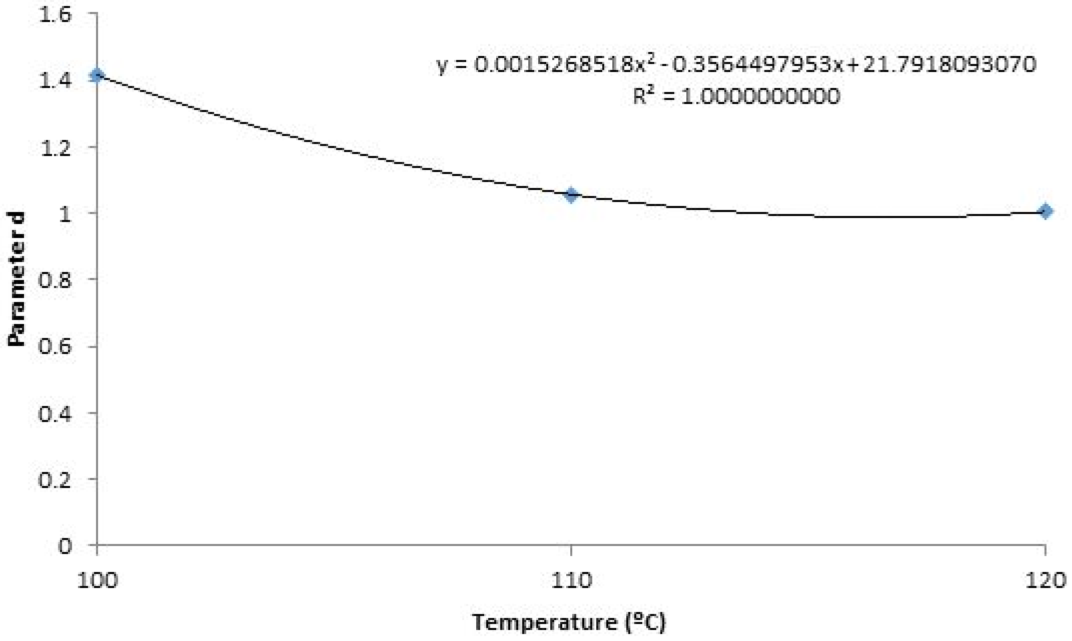

Figure S7. Parameter d for 40 bar with the temperature

Parameter d for 50 bar

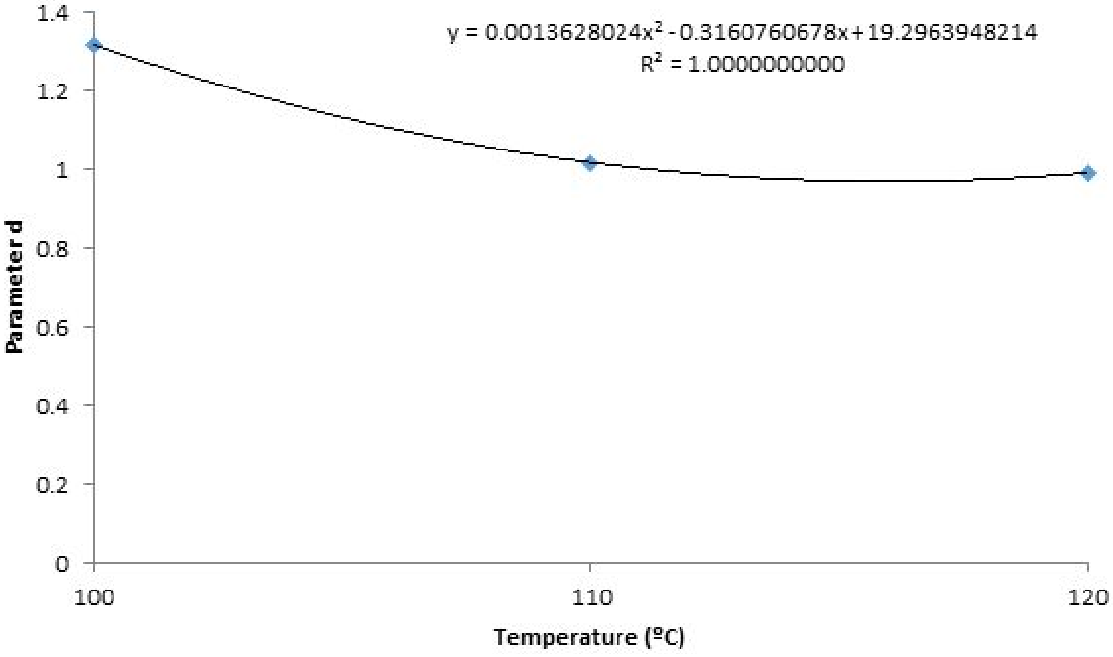

Figure S8. Parameter d for 50 bar with the temperature

Parameter d for 60 bar

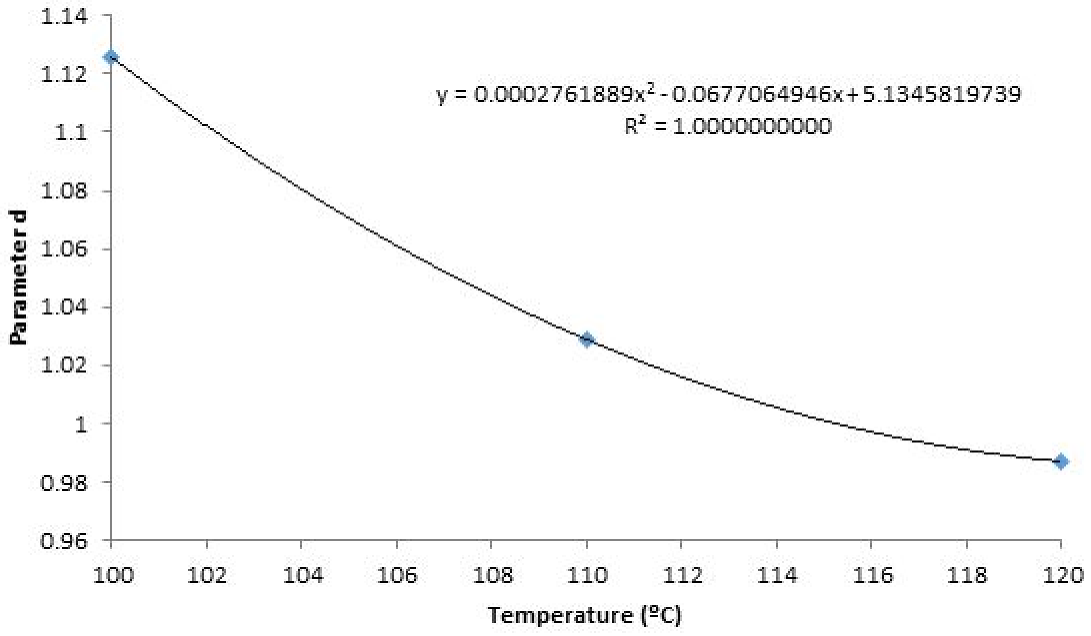

Figure S9. Parameter d for 60 bar with the temperature

Parameter e for 40 bar

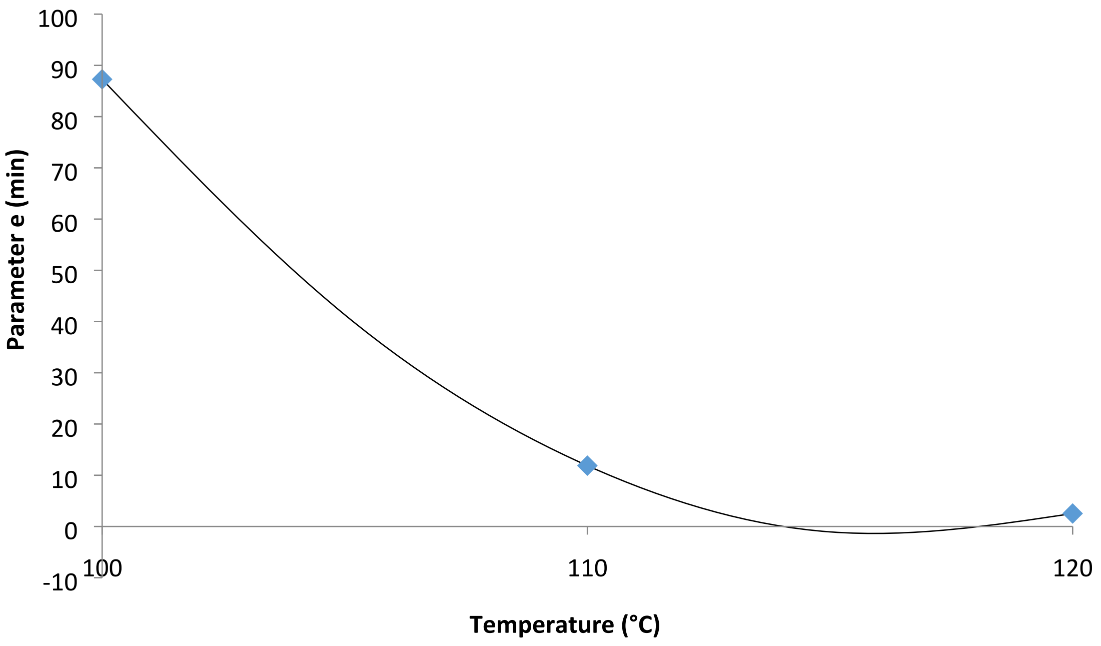

Figure S10. Parameter e for 40 bar with the temperature

Parameter e for 50 bar

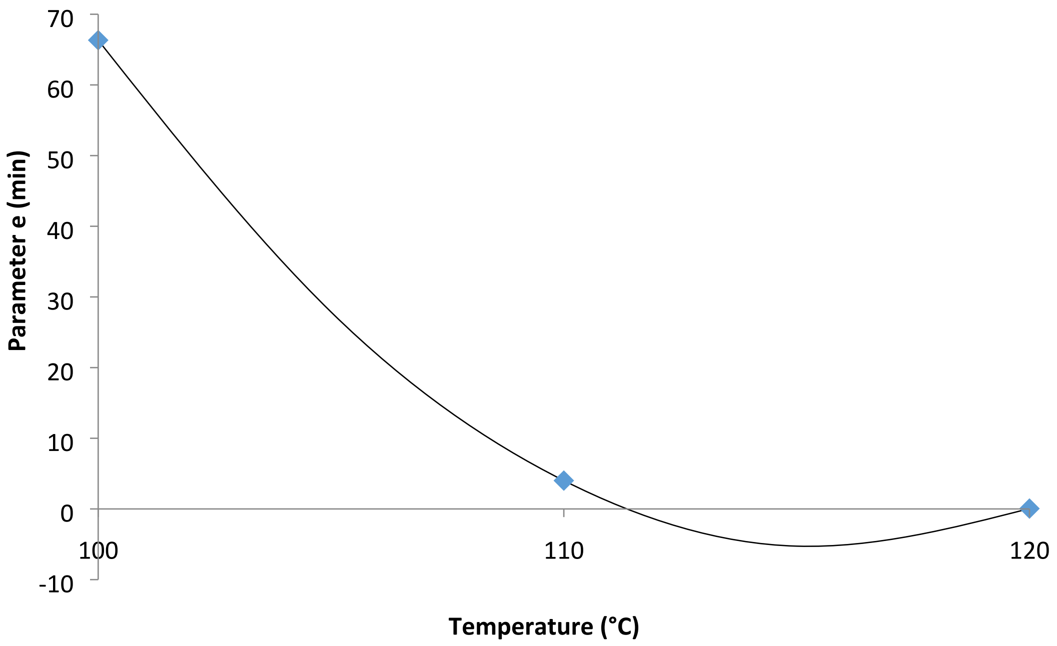

Figure S11. Parameter e for 50 bar with the temperature

Parameter e for 60 bar

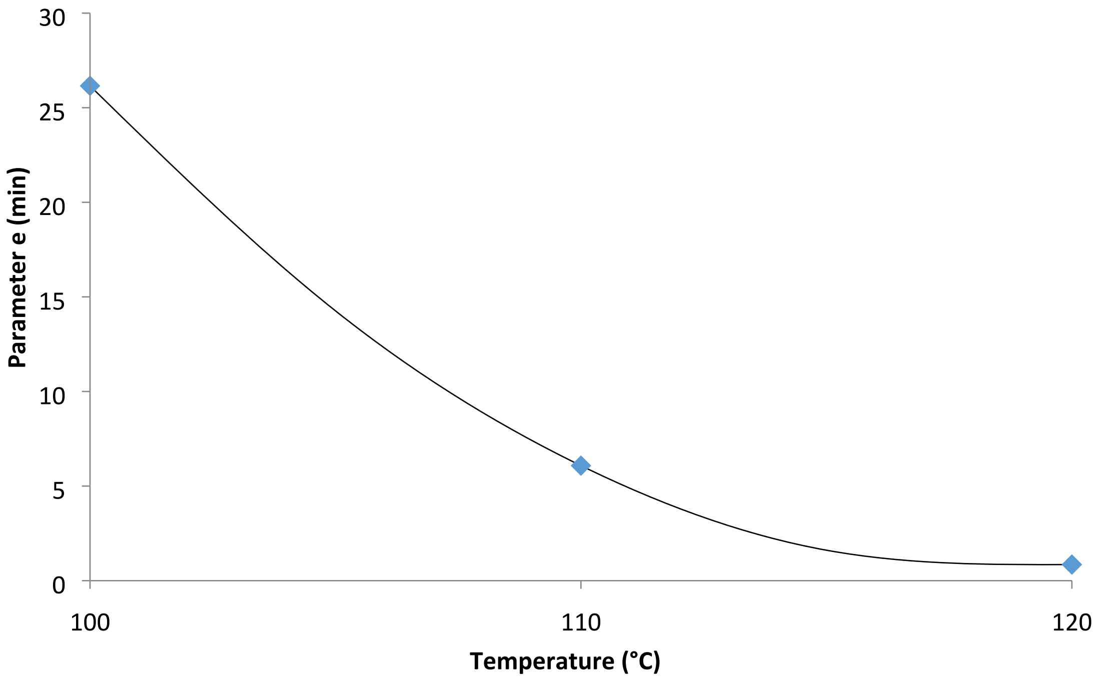

Figure S12. Parameter e for 60 bar with the temperature

Evaporators modeling

$$H_L = \sum l_i \cdot (\Delta \mathrm{H}_{form\, i} + \int_{T_{ref}}^{T} c_{pi} \cdot dT)$$

Where $L = \sum l_i$

The steam used in the first effect, HS, is a saturated steam.

$$H_S = S \cdot (\Delta \mathrm{H}_{form\ Wa(liq)} + \int_{T_{ref}}^{T_{eb}} c_{p,liq} \cdot dT + \lambda(\mathrm{T}_{eb}))$$

Hs is the enthalpy of the condensed steam and it¨s calculated as follows:

$$H_s = S \cdot (\Delta \mathrm{H}_{form\ Wa(liq)} + \int_{T_{ref}}^{T_{eb}} c_{p,liq} \cdot dT)$$

Economic evaluation of different biomasses

a) b)

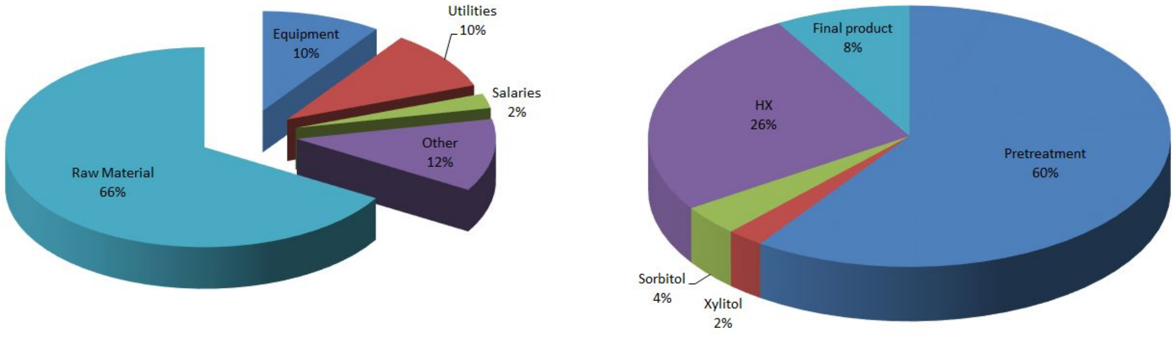

Figure S13. Dilute Acid- Catalytic Hydrogenation Corn Stover

a) b)

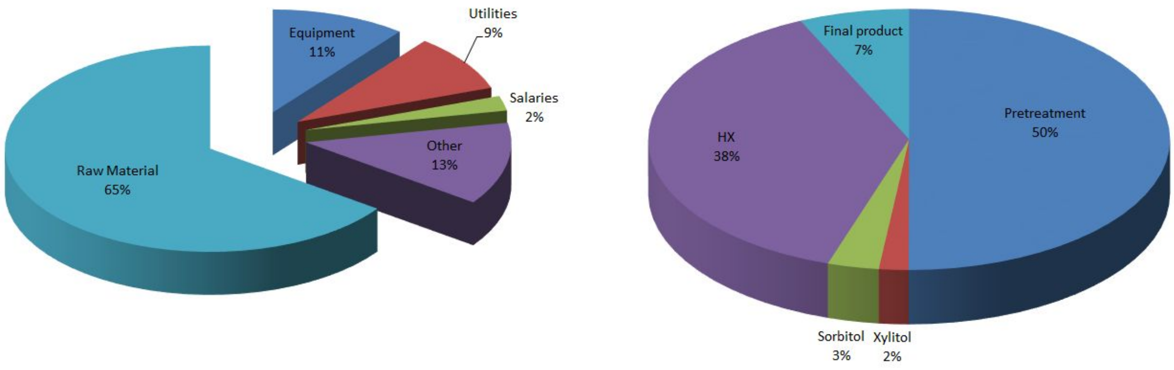

Figure S14. Dilute Acid- Catalytic Hydrogenation Birch

a) b)

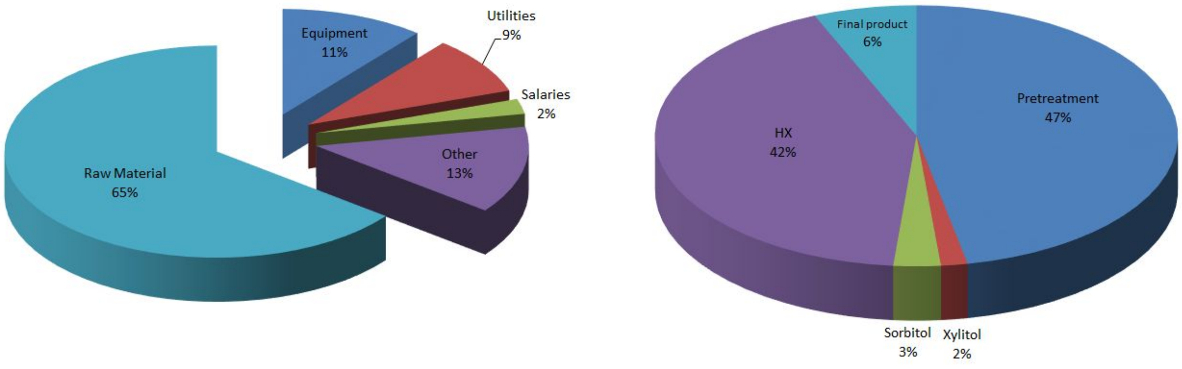

Figure S15. Dilute Acid- Catalytic Hydrogenation Pine

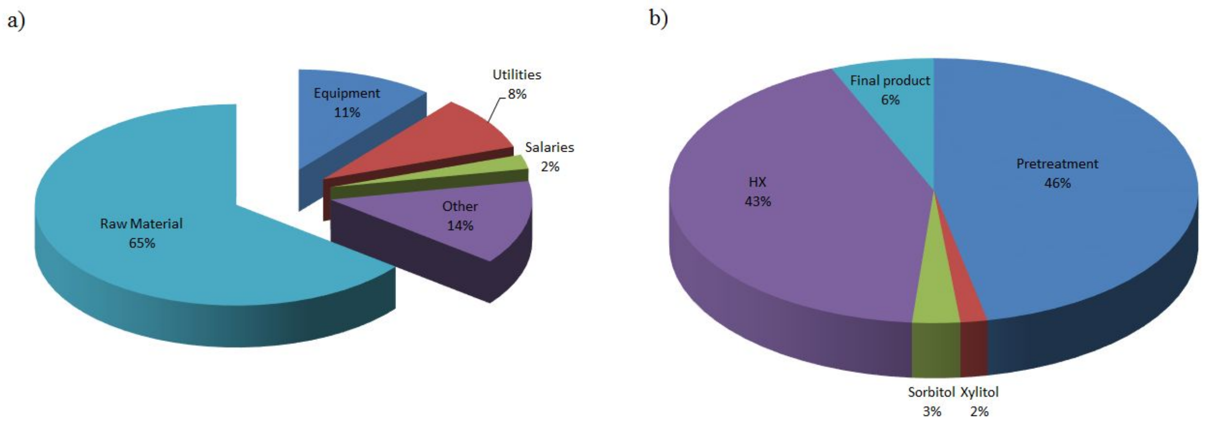

Figure S16. Dilute Acid- Catalytic Hydrogenation Spruce

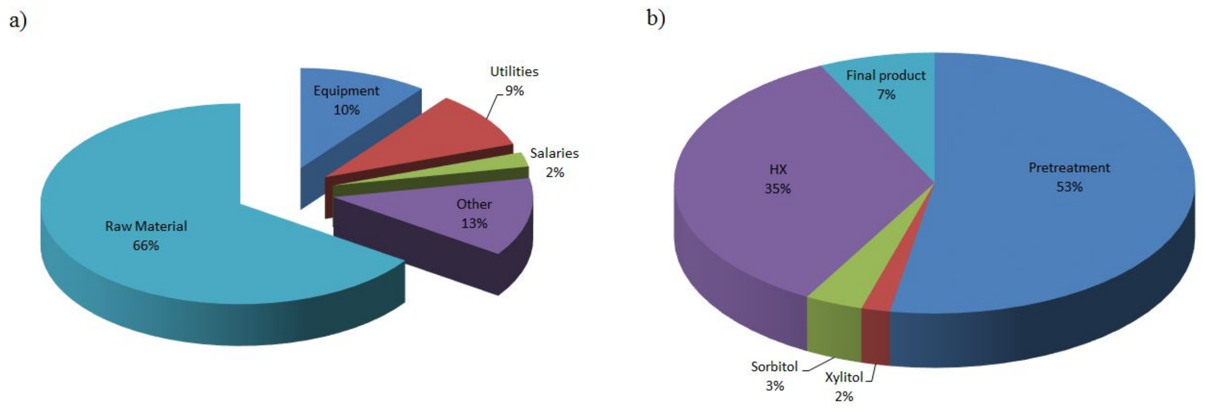

Figure S17. Dilute Acid- Catalytic Hydrogenation Poplar

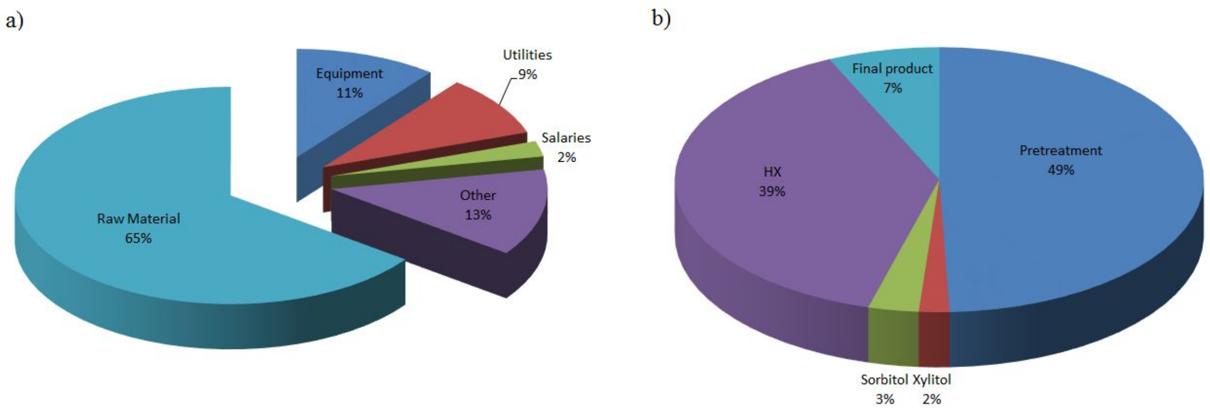

Figure S18. Dilute Acid- Catalytic Hydrogenation Sugarcane Bagasse

a) b)

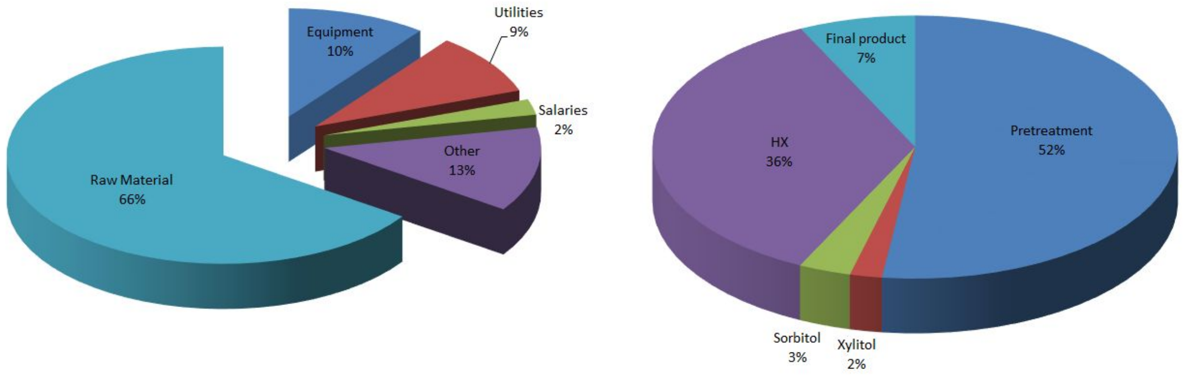

Figure S19. Dilute Acid- Catalytic Hydrogenation Wheat Straw
